# The mechanisms of DEPDC7-mediated actin cap establishment and meiosis progression in oocytes

**DOI:** 10.1038/s44319-026-00842-0

**Published:** 2026-06-22

**Authors:** Jian Mu, Siyuan Xie, Lin Zhao, Yang Zeng, Hao Gu, Zhiqi Pan, Ran Yu, Zhihua Zhang, Tianyu Wu, Weijie Wang, Lei Wang, Qing Sang

**Affiliations:** 1 Institute of Pediatrics, Children’s Hospital of Fudan University, State Key Laboratory of Genetic Engineering, Institutes of Biomedical Sciences, Shanghai Key Laboratory of Medical Epigenetics, Fudan University, Shanghai, 200032 China; 2 Department of Bioengineering, University of Pennsylvania, Philadelphia, 19104 PA USA; 3 Center for Reproductive Medicine, Department of Gynecology & Obstetrics, the First Affiliated Hospital, Sun Yat-Sen University, Guangzhou, 510080 China; 4 Guangdong Provincial Key Laboratory of Reproductive Medicine, Guangzhou, 510080 China; 5 Guangdong Provincial Clinical Research Center for Obstetrical and Gynecological Diseases, Guangzhou, 510080 China; 6 International Peace Maternity and Child Health Hospital, School of Medicine, Shanghai Jiao Tong University, Shanghai, 200030 China; 7 Shanghai Academy of Natural Sciences (SANS), Fudan University, Shanghai, China

**Keywords:** Cell Adhesion, Polarity & Cytoskeleton, Cell Cycle, Development

## Abstract

Asymmetric division is crucial for oocyte and embryo development. During meiosis I, the spindle migrates to the subcortex, and F-actin accumulates to form the actin cap, which supports polar body extrusion. While the CDC42–NWASP–ARP2/3 pathway, regulated by Ran-GTP, is known to promote actin cap formation, how Ran-GTP controls cortical polarization and the precise function of the actin cap remain unclear. Here, we identify *Depdc7* as a novel actin cap–localized protein essential for actin cap formation and first polar body extrusion. DEPDC7 is recruited to the oocyte membrane by Ran-GTP and acts as a scaffold to promote cortical actin assembly. Strikingly, inhibiting CDK1 activity partially rescues the extrusion defect in *Depdc7*-depleted oocytes. These findings indicate that both CDK1 activity and actin cap formation are essential for polar body extrusion and function synergistically. Our study refines the molecular mechanism of actin cap assembly and highlights DEPDC7 as a key regulator of oocyte maturation and female fertility.

## Synopsis

This study identifies DEPDC7 as a novel scaffold protein recruited by Ran-GTP to orchestrate actin cap formation during oocyte meiosis I. These findings reveal that DEPDC7-mediated cortical actin assembly acts synergistically with CDK1 inactivation to ensure successful first polar body extrusion and female fertility.

- Deletion of Depdc7 in mice leads to failure of first polar body extrusion, resulting in female infertility.
- Ran-GTP recruits DEPDC7 to the oocyte cortex, where it promotes cortical actin cap assembly.
- First polar body extrusion requires coordinated action of the actin cap and CDK1 regulation, whereas second polar body extrusion can occur independently of the actin cap.

## Abstract (web summary)

This study identifies DEPDC7 as a novel scaffold protein recruited by Ran-GTP to orchestrate actin cap formation during oocyte meiosis I. These findings reveal that DEPDC7-mediated cortical actin assembly acts synergistically with CDK1 inactivation to ensure successful first polar body extrusion and female fertility.

## Graphical abstract

**Figure:**
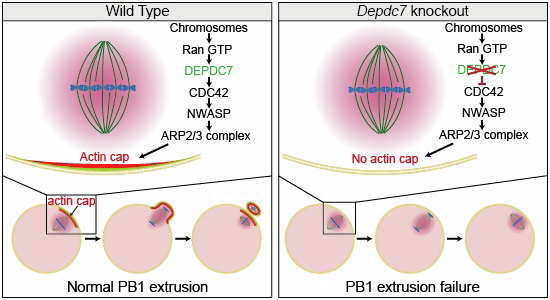

**Figure:**
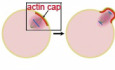

## Introduction

Asymmetric division is one of the most prominent features of oocyte meiosis. In this process, the extrusion of a small polar body ensures the retention of most of the cytoplasmic components in the oocyte, which is essential for fertilization and early embryo development. Asymmetric division is driven by the eccentric positioning of the meiotic spindle at the oocyte cortex (Azoury et al, 2008; Fabritius et al, 2011; Verlhac et al, 2000), and this process is regulated by actin cytoskeleton dynamics. The meiotic spindle is assembled after germinal vesicle (GV) breakdown, and formin-2-nucleated actin filaments (F-actin) begin to induce random motion of the spindle. The cytoplasmic actin network exerts forces on the spindle, allowing its motion to the cortex (Azoury et al, 2011; Schuh and Ellenberg, 2008; Yi et al, 2013). Once the spindle is close to the cortex, F-actin rapidly accumulates in the cortex to collapse the microvilli and form a thick actin layer referred to as the actin cap. The actin cap is important for spindle positioning, spindle rotation, and polar body extrusion (Dehapiot et al, 2013; Maro et al, 1986; Wang et al, 2020; Yi et al, 2011; Zhu et al, 2003), thus facilitating oocyte maturation. Therefore, gaining insights into oocyte actin cap formation is of great importance for understanding polar body extrusion and oocyte maturation.

Several studies focusing on the mechanism of actin cap formation have shown that this process is regulated by the CDC42-NWASP-ARP2/3 complex pathway. Activated CDC42-GTP activates NWASP, which in turn activates the ARP2/3 complex, and the activated ARP2/3 complex promotes actin cap formation (Dehapiot et al, 2013; Yi et al, 2011). Blocking this pathway leads to failure of actin cap formation. The Ran-GTPase gradient that emanates from chromosomes is also a key signal for actin cap formation (Deng et al, 2007; Yi et al, 2011), and overexpression of the dominant Ran-T24N mutation in oocytes causes defective actin cap formation (Deng et al, 2007). It is generally believed that Ran-GTP is the upstream signal regulating the CDC42-NWASP-ARP2/3 complex pathway (Dehapiot and Halet, 2013; Deng et al, 2007; Namgoong and Kim, 2016). Moreover, Ran-GTP also controls ERM (Ezrin/Radixin/Moesin) dephosphorylation, which mediates the collapse of the microvilli in the polarized cortex (Dehapiot and Halet, 2013). However, despite the above findings, it is still unclear how the Ran-GTP signal is transduced to the CDC42-NWASP-ARP2/3 pathway, and the earlier effectors downstream of Ran-GTP that regulate actin accumulation and actin cap formation remain to be identified.

Here, we identified DEPDC7 as a novel actin cap-localized protein and found that female mice lacking *Depdc7* are completely infertile. DEPDC7 serves as an early effector that responds to Ran-GTP signaling, and it anchors to the plasma membrane to initiate F-actin accumulation and actin cap formation. *Depdc7* deletion prevents the formation of the actin cap, thus disrupting the extrusion of the first polar body (PB1). Unexpectedly, the arrested oocyte can still be fertilized and extrude the second polar body, leading to the formation of triploid embryos and female infertility. Finally, we found that inhibiting CDK1 activity allows *Depdc7*-deleted oocytes to extrude PB1. Our findings indicate the specific role of *Depdc7* in regulating oocyte actin cap formation and meiosis progression and reveal a mechanistic difference in polar body extrusion between meiosis I and meiosis II.

## Results

### Identification of DEPDC7 as a novel oocyte actin cap-localized protein

**Figure EV1:**
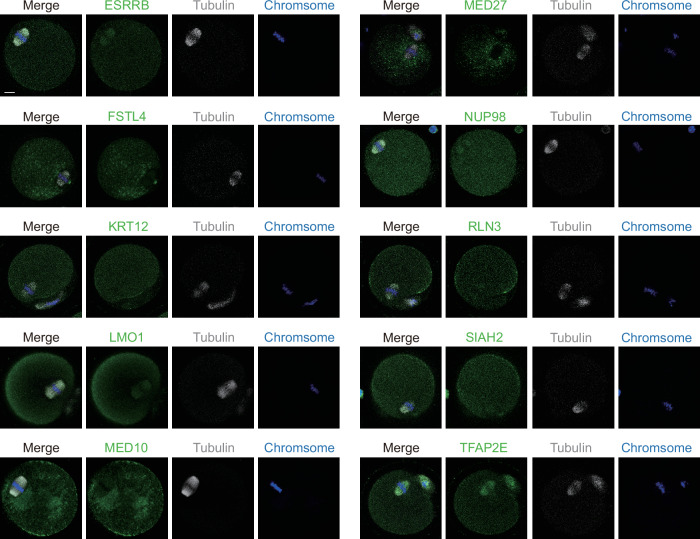
Immunofluorescence staining of the indicated protein in oocytes. The protein shows high expression in the oocyte. blue, Hoechst-labeled chromosome; gray, FITC-tubulin–labeled microtubules; green, indicated proteins. Scale bar, 10 μm. Source data are available online for this figure.

**Figure 1:**
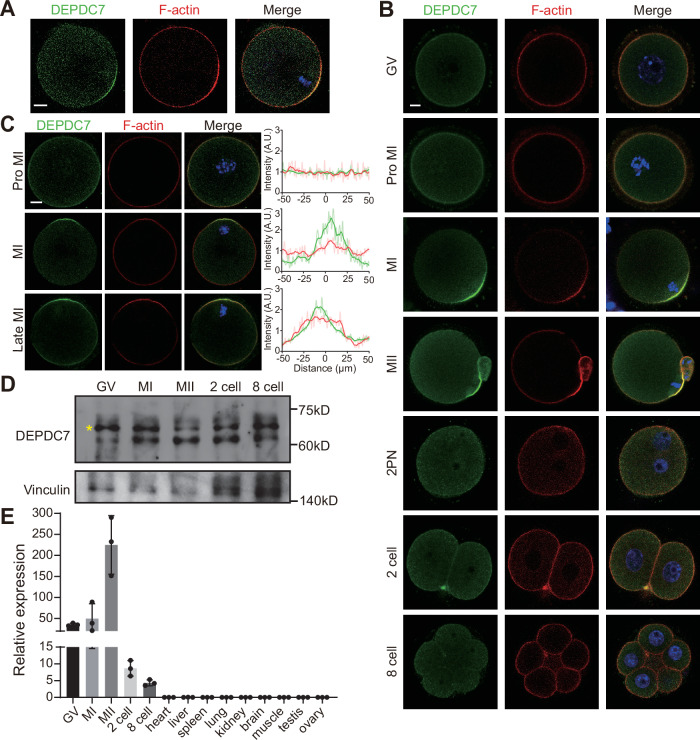
DEPDC7 co-localizes with cortical F-actin during oocyte maturation. (**A**) Expression and localization of *Depdc7* in MI oocyte (*n* = 12). Red, phalloidin-labeled F-actin; blue, Hoechst-labeled DNA; green, DEPDC7. Scale bar, 10 μm. (**B**) Expression and localization of DEPDC7 from GV to the eight-cell stage. Red, phalloidin-labeled F-actin; blue, Hoechst-labeled DNA; green, DEPDC7. Scale bar, 10 μm. (**C**) Immunofluorescence images of different stages of MI oocyte. Red, phalloidin-labeled F-actin; blue, Hoechst-labeled DNA; green, DEPDC7. The measurements of F-actin (red) and DEPDC7 (green) intensity were generated along the oocyte perimeter are shown at the right. Scale bar, 10 μm. For normalization, the mean fluorescence intensity along the entire line-scan was set to 1, and the fluorescence intensity at each point was normalized to this value. (**D**) Immunoblot of DEPDC7 and vinculin from GV to the eight-cell stage. Sixty oocytes or embryos were collected from each period for immunoblotting, and the results were repeated three times. The yellow star indicates an unspecific band. (**E**) Comparison of *Depdc7* expression patterns in mice by qRT-PCR, normalized to *Gapdh*. *n* = 3 independent biological replicates. Primers used for qRT-PCR are listed in the Reagents and Tools Table. Data were presented as mean ± SD. Source data are available online for this figure.

Previously, we performed immunofluorescence for a group of proteins with relatively high expression in oocytes, but their roles in oocyte development are rarely explored (Fig. EV1). And we found that DEPDC7 showed specific localization in the actin cap of metaphase I (MI) and metaphase II (MII) oocytes (Fig. 1A). This localization prompted us to speculate that DEPDC7 may play essential roles in the formation of the actin cap and oocyte maturation.

We first focused on the expression and localization patterns during oocyte and early embryonic development. At the GV stage, DEPDC7 was weakly and evenly distributed in the oocyte cortex (labeled by phalloidin). This localization pattern was maintained to the MI stage (~6 h after NEBD) when the spindle was assembled in the center of the oocyte. However, as the spindle migrated near the oocyte cortex at the MI stage (~8 h after NEBD), DEPDC7 was preferentially enriched in the actin cap region. The actin cap location of DEPDC7 was also observed in metaphase II (MII) oocytes, but rapidly disappeared after fertilization (Fig. 1B). We next examined the accumulation of DEPDC7 and F-actin in the polarity region during the MI stage. Immunofluorescence staining revealed that DEPDC7 underwent polar localization earlier than F-actin at the MI stage (Fig. 1C). At the protein level, DEPDC7 increased gradually from GV to MII but rapidly decreased from the 2-cell stage (Fig. 1D). To confirm the specificity of the DEPDC7 band, *mDepdc7* was overexpressed in oocytes. Upon overexpression, the expected DEPDC7 band was specifically restored, whereas the additional band remained unchanged, indicating that it represents a non-specific signal (Appendix Fig. S1). Importantly, *Depdc7* was highly expressed in oocytes compared with other tissues (Fig. 1E), showing a relatively oocyte-specific expression pattern. Collectively, we identified a novel actin cap-localized protein DEPDC7, which may play an important role in oocyte maturation.

### *Depdc7* deficiency leads to female infertility by disrupting PB1 extrusion

**Figure EV2:**
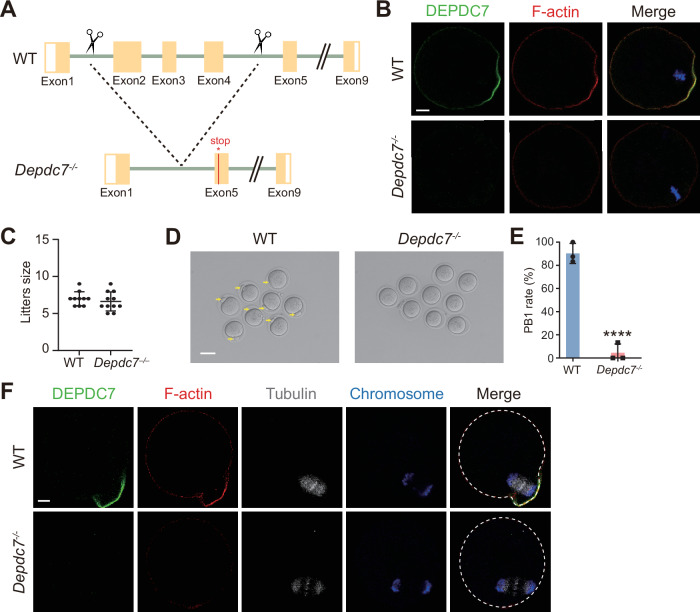
Oocyte from *Depdc7*^*–/–*^ mice could not extrude PB1. (**A**) Schematic diagram of the genome of the *Depdc7* knockout mouse. Exons 2–4 were knocked out, leading to premature termination in exon 5. (**B**) Immunofluorescence staining of ovulated oocytes from WT (*n* = 35) and *Depdc7*^–/–^ (*n* = 29) mice. Red, phalloidin-labeled F-actin; blue, Hoechst-labeled DNA; green, DEPDC7. Scale bar, 10 μm. (**C**) Fertility assay of WT (*n* = 10) and *Depdc7*^–/–^ (*n* = 11) male mice. Data were presented as mean ± SD. (**D**) Brightfield image of WT (*n* = 40) or *Depdc7*^–/–^ (*n* = 30) oocytes during in vitro maturation. The yellow arrow points to PB1. Scale bar, 50 μm. (**E**) The PB1 extrusion rate in (**D**). Data were presented as mean ± SD. *P* = 2.05 × 10^−9^, Fisher’s exact test. (**F**) Immunofluorescence images of AI (anaphase I) in WT (*n* = 15) and *Depdc7*^–/–^ (*n* = 13) oocytes. Red, phalloidin-labeled F-actin; blue, Hoechst-labeled chromosome; gray, FITC-tubulin–labeled microtubules; green, DEPDC7. Dashed lines indicate the oocyte boundary. Scale bar, 10 μm. *n* indicates the number of oocytes analyzed. All experiments were performed in at least three independent biological replicates. Source data are available online for this figure.

**Figure 2:**
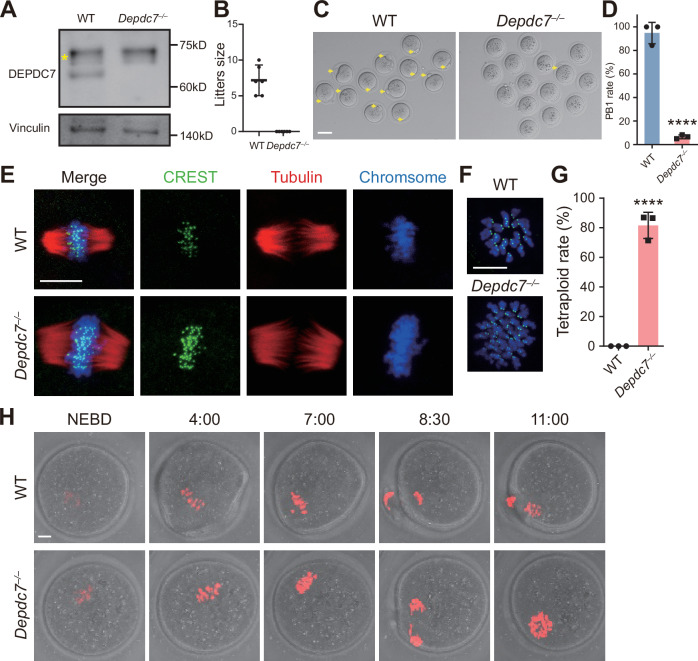
*Depdc7* is essential for polar body extrusion and female fertility. (**A**) Immunoblot of DEPDC7 and vinculin in MII oocytes from WT and *Depdc7*^–/–^ mice. Sixty oocytes were collected from each group for immunoblotting, and the results were repeated three times. The yellow star indicates an unspecific band. (**B**) Fertility assay of WT (*N* = 6) and *Depdc7*^–/–^ (*N* = 6) female mice. (**C**) Brightfield images of oocytes that were retrieved from the oviducts of WT (*n* = 39) and *Depdc7*^–/–^ (*n* = 59) mice through hormonal stimulation. The yellow arrow points to PB1. Scale bar, 50 μm. (**D**) The PB1 extrusion rate in (**C**). *P* = 8.51 × 10^−19^, Fisher’s exact test. (**E**) Immunofluorescence images of CREST (green), tubulin (red), and chromosomes (blue) in WT (*n* = 18) and *Depdc7*^–/–^ oocytes (*n* = 23). Scale bar, 10 μm. (**F**) Chromosome and centromere staining of ovulated oocytes from WT (*n* = 17) and *Depdc7*^–/–^ (*n* = 52) mice. Scale bar, 10 μm. (**G**) Quantification of centromere number in (**F**). *P* = 5.80 × 10^−11^, Fisher’s exact test. (**H**) Representative time-lapse images showing chromosome dynamics during WT (*n* = 15) and *Depdc7*^–/–^ oocyte (*n* = 12) maturation after NEBD. Red, H2B-mScarlet-labeled chromosomes. Scale bar, 10 μm. Data were presented as mean ± SD. *n* indicates the number of oocytes analyzed. All experiments were performed in at least three independent biological replicates. Source data are available online for this figure.

To investigate the function of *Depdc7* in oocytes, we generated *Depdc7* knockout mice (*Depdc7*^–/–^) by deleting exons 2–4 (Fig. EV2A). DEPDC7 protein was completely undetectable in *Depdc7*^–/–^ oocytes as confirmed by immunofluorescence and immunoblotting (Figs. 2A and EV2B). No obvious morphological or behavioral abnormalities were observed in *Depdc7*^–/–^ mice, and *Depdc7*^–/–^ male mice were fertile (Fig. EV2C). However, *Depdc7*^–/–^ female mice were completely infertile after mating with wild-type (WT) males for 6 months (Fig. 2B), indicating an essential role of DEPDC7 in female reproduction.

To explore the reason for the infertility of *Depdc7*^–/–^ female mice, oocytes were super-ovulated through hormonal stimulation. Although comparable numbers of oocytes were recruited from *Depdc7*^–/–^ female mice, the majority of them did not extrude a first polar body (PB1) (Fig. 2C,D). This phenotype was confirmed by an in vitro maturation assay using GV oocytes (Fig. EV2D,E), showing the typical phenotype of oocyte maturation arrest. Immunofluorescence showed that these *Depdc7*^–/–^ oocytes were arrested at metaphase but exhibited a larger spindle volume with a greater number of chromosomes and centromeres than WT oocytes (Fig. 2E). Centromere counting showed that the ovulated *Depdc7*^–/–^oocytes were in a tetraploid state (Fig. 2F,G). These results prompted us to determine whether the oocytes were arrested at MI or, as previously reported in *Fmn2* (formin-2) deficiency oocyte (Dumont et al, 2007), had undergone anaphase I (AI), followed by separation of homologous chromosomes and their reassembly into a unique MII spindle. To track the dynamic changes in chromosomes, we performed time-lapse imaging of in vitro-cultured GV oocytes that expressed H2B-mScarlet. Unexpectedly, the *Depdc7*^–/–^ oocytes experienced the AI stage as characterized by the segregation of homologous chromosomes at about 8.5 h after NEBD (Fig. 2H; Movies EV1 and EV2). Furthermore, we performed higher temporal resolution live imaging of oocytes at 6.5 h after NEBD, acquiring images every 2 min to monitor the AI progression in oocytes. In WT oocytes, following anaphase onset, homologous chromosomes segregate rapidly, accompanied by membrane protrusion, resulting in the extrusion of the PB1. In *Depdc7*^–/–^ oocytes, although homologous chromosomes also segregate promptly upon anaphase onset, the spindle moves within the cytoplasm, indicating that it cannot stably anchor at the cortex in the absence of an actin cap. Notably, one set of chromosomes remained adjacent to the plasma membrane without triggering membrane protrusion, and after a period, the segregated homologous chromosomes reassembled into a new spindle. (Movies EV3 and EV4). However, due to the failure of PB1 extrusion the segregated chromosomes were reunited and the spindle was reassembled, thus leading to the formation a seemingly MII-stage oocyte. This phenomenon was also confirmed by immunofluorescence in fixed oocytes, showing that the chromosomes of *Depdc7*^–/–^ oocytes could separate normally as in WT oocytes, but the cortical membrane protrusion did not occur (Fig. EV2F). Taken together, our findings show that *Depdc7*^–/–^ oocytes are not arrested in MI. They undergo AI, after which the separated homologous chromosomes are reassembled on the metaphase II plate, as described before for *Fmn2* deficiency oocytes (Dumont et al, 2007). *Depdc7*^–/–^ oocytes are thus arrested in metaphase II while retaining both sets of homologous chromosomes due to failure of PB1 extrusion.

### DEPDC7 is essential for oocyte actin cap formation

**Figure 3:**
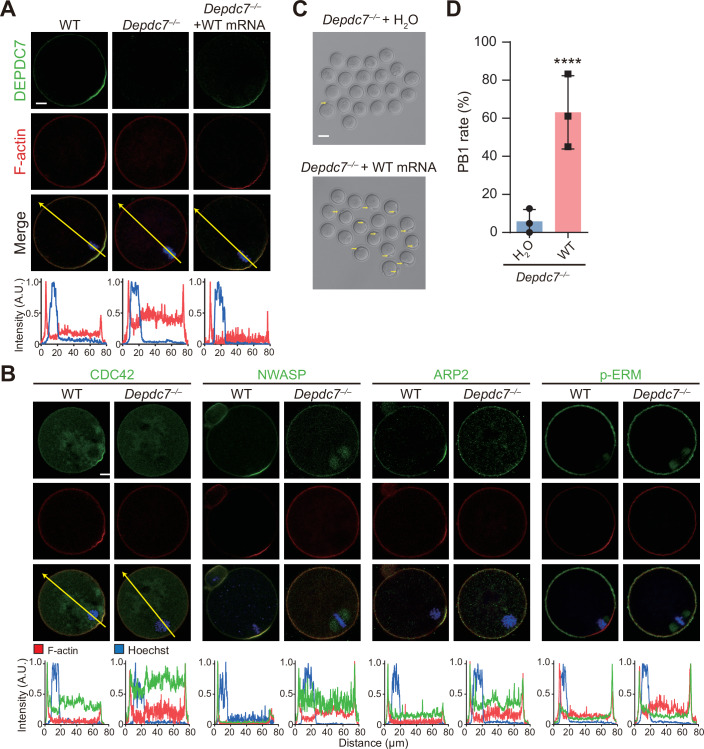
DEPDC7 regulates actin cap formation and PB1 extrusion. (**A**) Immunofluorescence images of DEPDC7 and F-actin in WT oocytes (*n* = 23), *Depdc7*^–/–^ oocytes (*n* = 30), and *Depdc7*^–/–^ oocytes overexpressing *Depdc7* mRNA (*n* = 20). Red, phalloidin-labeled F-actin; blue, Hoechst-labeled DNA; green, DEPDC7. Scale bar, 10 μm. The measurements of F-actin (red) and chromosomes (blue) intensity along the direction of the yellow arrows are shown at the bottom. (**B**) Immunofluorescence images of CDC42, ARP2, NWASP, and p-ERM in WT (left) and *Depdc7*^–/–^ (right) oocytes. Red, phalloidin-labeled F-actin; blue, Hoechst-labeled DNA; green, target proteins. Scale bar, 10 μm. More than 20 oocytes were examined per group across three replicates. The measurements of target protein (green), chromosomes (blue) and F-actin (red) intensity along the direction of the yellow arrows are shown at the bottom. (**C**) Brightfield image of *Depdc7*^–/–^ oocytes injected with water (*n* = 51) or *Depdc7* mRNA (*n* = 59) during in vitro maturation. The yellow arrow points to PB1. Scale bar, 50 μm. (**D**) The PB1 extrusion rate in (**C**). *P* = 9.01 × 10^−9^, Fisher’s exact test. Data were presented as mean ± SD. *n* indicates the number of oocytes analyzed. All experiments were performed in at least three independent biological replicates. For intensity normalization in (**A**, **B**), the minimum intensity value along the line-scan was set to 0, the maximum value was set to 1.0, and all intermediate values were scaled accordingly. Source data are available online for this figure.

Next, we asked why *Depdc7*^–/–^ oocytes failed to extrude PB1 even though the homologous chromosomes had been segregated. Because DEPDC7 has a typical location in the oocyte actin cap, we first evaluated the influence of DEPDC7 depletion on oocyte actin cap formation. In WT oocytes, actin signals were enriched in the actin cap. However, in *Depdc7*^–/–^ oocytes, the actin signals were evenly distributed in the membrane and no actin cap region was observed by phalloidin staining (Fig. 3A). To confirm that the actin cap was absent in *Depdc7*^–/–^ oocytes, we further detected the localization of other relevant proteins involved in actin cap formation, such as CDC42, NWASP, and ARP2. Similarly, the immunofluorescence of all three proteins did not label a specific actin cap region (Fig. 3B). Also, actin cap formation is accompanied by ERM dephosphorylation in the polarized region, and we found that DEPDC7 deficiency resulted in the loss of the dephosphorylated ERM region (Fig. 3B). When we supplemented DEPDC7 in *Depdc7*^–/–^ oocytes by injecting *Depdc7* mRNA, the actin cap was constructed normally (Fig. 3A) and PB1 was extruded (Fig. 3C,D). To examine whether cytoplasmic actin was affected in *Depdc7*^–/–^ oocytes, we assessed F-actin using phalloidin staining. Cytoplasmic F-actin intensity was not reduced; rather, it showed a slight but statistically significant increase (Appendix Fig. S2), indicating that DEPDC7 deficiency specifically disrupts cortical actin polarization without affecting overall cytoplasmic F-actin dynamics. Together, these results demonstrated that *Depdc7* depletion resulted in the complete loss of the actin cap and the disruption of PB1 extrusion.

### DEPDC7 is an early effector downstream of Ran-GTP that regulates actin accumulation

**Figure 4:**
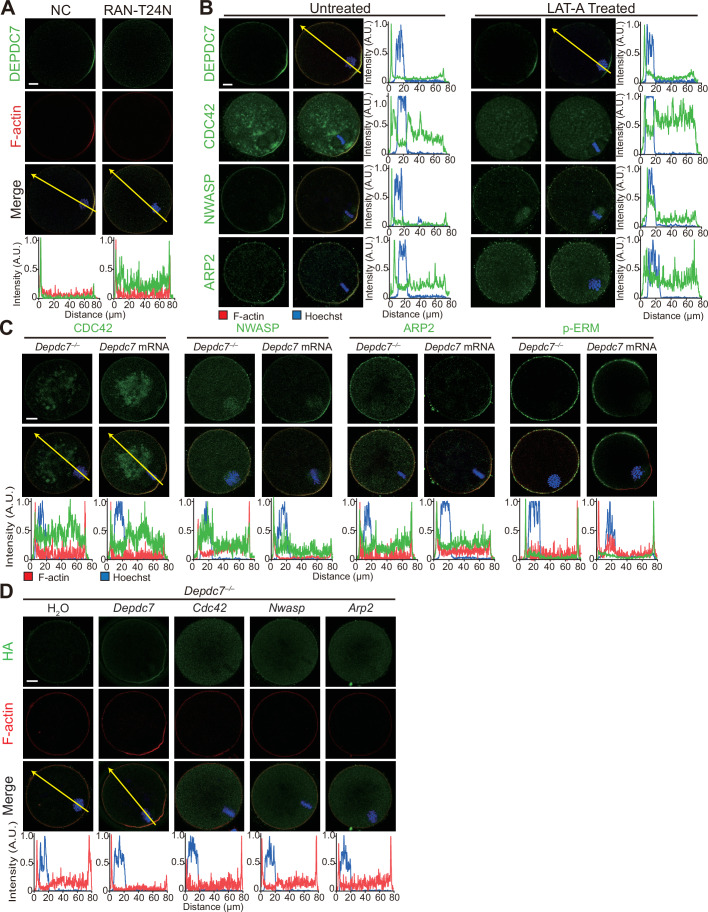
DEPDC7 is localized to the polar region and responds to Ran-GTP. (**A**) Location of DEPDC7 and F-actin in WT MII oocytes with (*n* = 25) or without (NC, *n* = 16) overexpression of RAN-T24N. Red, phalloidin-labeled F-actin; blue, Hoechst-labeled DNA; green, DEPDC7. Scale bar, 10 μm. The measurements of DEPDC7 (green) and F-actin (red) intensity along the direction of the yellow arrows are shown at the bottom. (**B**) Immunofluorescence images of WT MII oocytes treated with (right) or without (left) LAT-A. Red, phalloidin-labeled F-actin; blue, Hoechst-labeled DNA; green, target proteins. Scale bar, 10 μm. More than 25 oocytes were examined per group across three replicates. The measurements of target protein (green) and chromosomes (blue) intensity along the direction of the yellow arrows are shown at the right. (**C**) Immunofluorescence images of DEPDC7 and F-actin in *Depdc7*^–/–^ oocytes and *Depdc7*^–/–^ oocytes overexpressing *Depdc7* mRNA. Red, phalloidin-labeled F-actin; blue, Hoechst-labeled DNA; green, target proteins. Scale bar, 10 μm. More than 20 oocytes were examined per group across three replicates. The measurements of target protein (green), chromosomes (blue) and F-actin (red) intensity along the direction of the yellow arrows are shown at the bottom. (**D**) Immunofluorescence images of *Depdc7*^–/–^ oocytes overexpressing several mRNAs. Red, phalloidin-labeled F-actin; blue, Hoechst-labeled DNA; green, HA-tagged target proteins. Scale bar, 10 μm. More than 18 oocytes were examined per group across three replicates. The measurements of F-actin (red) and chromosomes (blue) intensity along the direction of the yellow arrows are shown at the bottom. All experiments were performed in at least three independent biological replicates. For intensity normalization, the minimum intensity value along the line-scan was set to 0 and the maximum value was set to 1.0, and all intermediate values were scaled accordingly. Source data are available online for this figure.

**Figure EV3:**
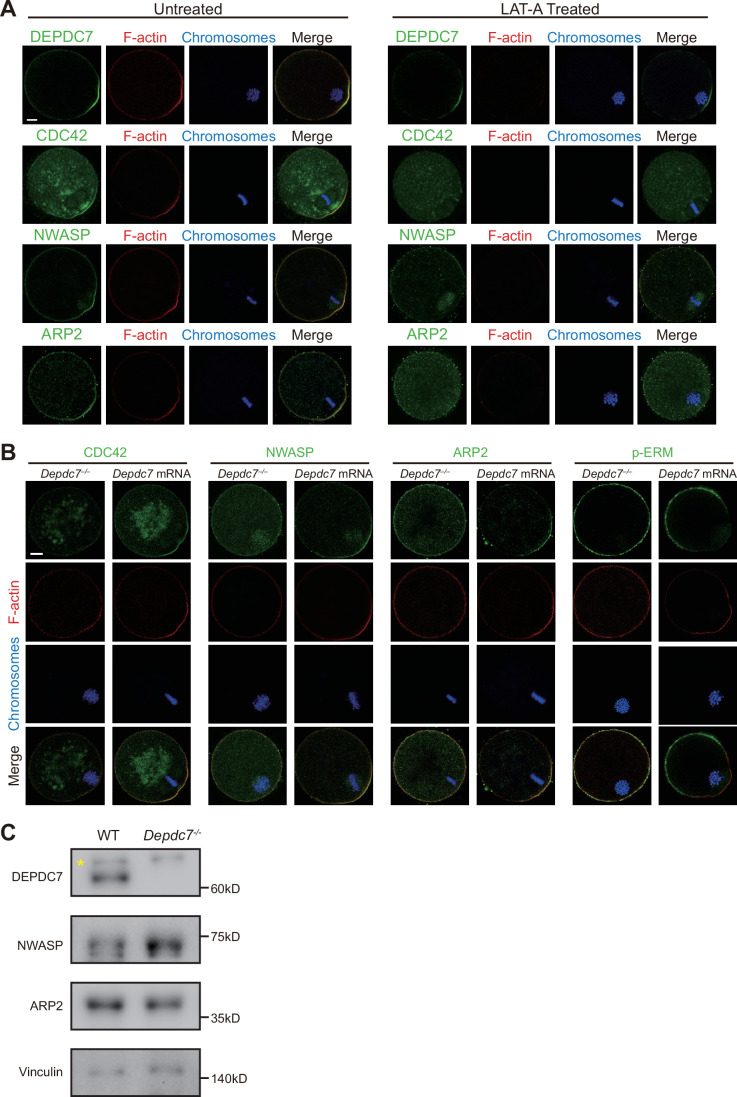
Depletion of DEPDC7 does not affect the expression of related proteins. (**A**,** B**) Full-channel images corresponding to Fig. 4B, C. (**C**) Immunoblot of DEPDC7, NWASP, ARP2, and vinculin in MII oocytes from WT and *Depdc7*^–/–^ mice. 60 oocytes were collected from each group for immunoblotting, and the results were repeated three times. The yellow star indicates an unspecific band. Source data are available online for this figure.

As shown in Fig. 1B, DEPDC7, like the actin cap, accumulates in the polarized region only when the spindle migrates to the subcortex. It has been reported that Ran-GTP acts as the upstream signal regulating the recruitment and activation of the Arp2/3 complex through the CDC42-NWASP pathway, thus promoting actin cap formation in oocytes. To determine whether the localization of DEPDC7 is also regulated by the Ran-GTP signal, we overexpressed dominant-negative Ran-T24N in WT oocytes, which disrupted the Ran-GTP gradient. The results showed that loss of Ran-GTP prevented the actin cap localization of DEPDC7 (Fig. 4A), suggesting that DEPDC7 is downstream of the Ran-GTP pathway. Because the CDC42-NWASP-ARP2/3 complex is also downstream of Ran-GTP, we investigated the mutual relationship between DEPDC7 and the CDC42-NWASP pathway. We treated WT oocytes with LAT-A (an inhibitor of G-actin polymerization), which led to F-actin depolymerization. After treatment with LAT-A, the localization of CDC42, ARP2, and NWASP was not seen in the polarized region, and the F-actin in the polarized region was also disrupted (Figs. 4B and EV3A). However, DEPDC7 still maintained its localization in the polarized region (Fig. 4B). Furthermore, the supplementation of DEPDC7 in *Depdc7*^–/–^ oocytes rescued the localization of CDC42, NWASP, and ARP2, and the ERM was dephosphorylated in the actin cap region (Figs. 4C and EV3B). However, the supplementation of CDC42, NWASP, and ARP2 in *Depdc7*^–/–^ oocytes could not rescue the formation of the actin cap, as indicated by the loss of the phalloidin signal (Fig. 4D). In addition, DEPDC7 depletion did not significantly influence the protein levels of NWASP or ARP2 in oocytes (Fig. EV3C). These results suggested that DEPDC7 acts as an early effector downstream of Ran-GTP and upstream of the CDC42-NWASP-ARP2/3 pathway to regulate actin cap formation.

### DEPDC7 is anchored to the plasma membrane and forms a scaffold for F-actin accumulation

**Figure 5:**
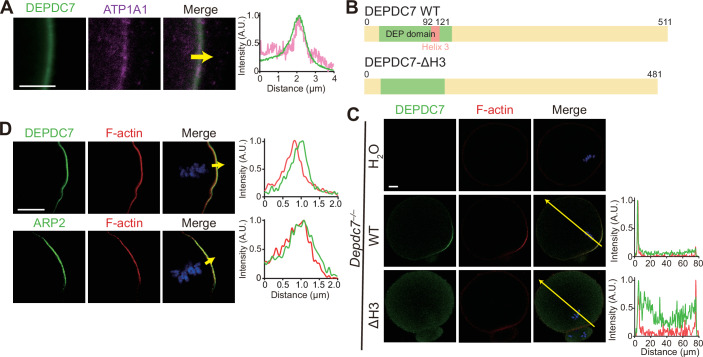
DEPDC7 is a membrane-localized protein. (**A**) Immunofluorescence images of mouse oocytes overexpressing *Depdc7* mRNA (*n* = 23). Magenta, ATP1A1; Green, DEPDC7. Scale bar, 5 μm. The measurements of DEPDC7 (green) and ATP1A1 (magenta) intensity along the direction of the yellow arrows are shown at the right. (**B**) Schematic showing the deletion of H3 in DEPDC7. (**C**) Immunofluorescence images of *Depdc7*^–/–^ oocytes injected with water (upper, *n* = 14), *Depdc7* WT mRNA (middle, *n* = 24), and *Depdc7*-ΔH3 mRNA (lower, *n* = 21). The measurements of F-actin (red) and DEPDC7 (green) intensity along the direction of the yellow arrows are shown at the right. Scale bar, 10 μm. (**D**) High-resolution immunofluorescence images of DEPDC7 (upper, *n* = 20) and ARP2 (lower, *n* = 23) with F-actin. Red, phalloidin-labeled F-actin; blue, Hoechst-labeled DNA; green, DEPDC7 or ARP2. Scale bar, 10 μm. The measurements of F-actin (red) and DEPDC7 or ARP2 (green) intensity along the direction of the yellow arrows are shown at the right. n indicates the number of oocytes analyzed. All experiments were performed in at least three independent biological replicates. For intensity normalization in (**A**, **C**, **D**), the minimum intensity value along the line-scan was set to 0, the maximum value was set to 1.0, and all intermediate values were scaled accordingly. Source data are available online for this figure.

Next, we explored how DEPDC7 generates polar signals independent of the actin cap. Because the DEPDC7 protein possesses a DEP domain, and it has been reported that the DEP domain can anchor the corresponding protein to the plasma membrane in cell lines (Consonni et al, 2014; Falginella et al, 2022), we verified whether DEPDC7 is localized to the membrane in oocytes. Immunofluorescence showed that DEPDC7 was completely co-localized with ATP1A1, which is a typical membrane protein marker (Fig. 5A), suggesting that DEPDC7 is localized to the membrane in oocytes. We then constructed a *Depdc7* mutant with deletion of helix 3 in the DEP domain (ΔH3) (Fig. 5B), which was predicted to disrupt the membrane binding ability of the DEP domain. Compared with overexpression of WT mRNA, overexpression of *Depdc7-*ΔH3 mRNA in *Depdc7*^–/–^ oocytes showed that most of the mutant protein was diffused in the cytoplasm rather than localized on the membrane (Fig. 5C), suggesting that DEPDC7 is anchored to the plasma membrane via its DEP domain. In contrast, high-resolution immunoimaging showed that DEPDC7 and F-actin were not completely co-localized in the polarized cap region. F-actin localized in an inner position beneath the DEPDC7 signal, whereas ARP2 was completely co-localized with F-actin (Fig. 5D). In addition, DEPDC7 underwent polar localization earlier than F-actin at the MI stage (Fig. 1C). These results indicated that DEPDC7 is anchored to the plasma membrane as a scaffold protein and responds earlier in response to the Ran-GTP signal and polarizes to the cortical region, where it then recruits F-actin to the membrane to form the oocyte actin cap.

### *Depdc7*^*–/–*^ oocytes have normal cytoplasmic maturation capacity

**Figure 6:**
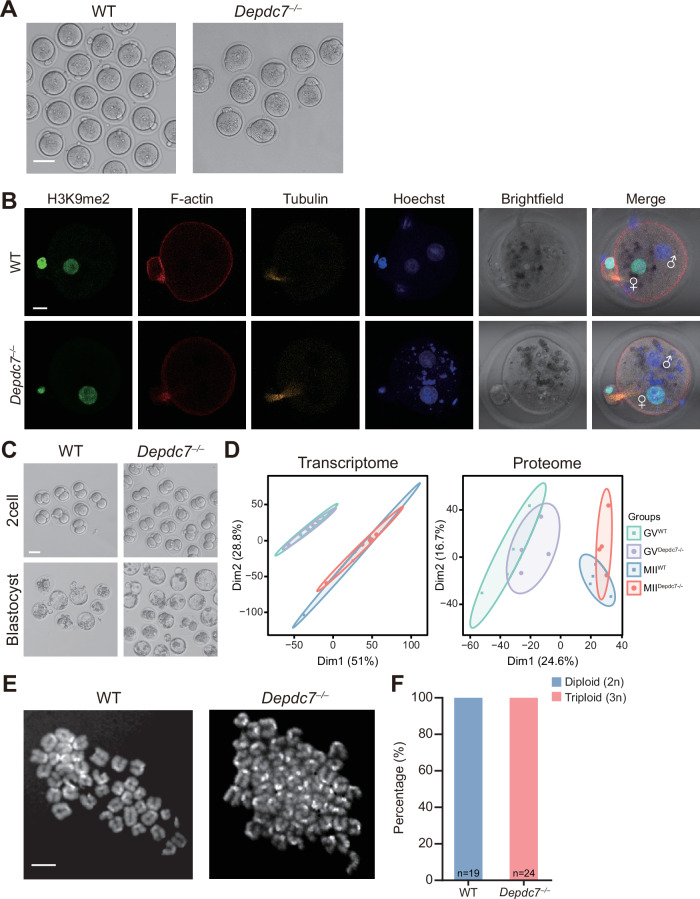
*Depdc7*^*–/–*^ oocytes have normal potential for fertilization and development. (**A**) Brightfield images of 2PN zygotes from WT (*n* = 58) and *Depdc7*^–/–^ (*n* = 60) mice after IVF. Scale bar, 50 μm. (**B**) Immunofluorescence images of 2PN zygotes from WT and *Depdc7*^–/–^ mice after IVF. ♂ male pronucleus; ♀ female pronucleus. Red, phalloidin-labeled F-actin; blue, Hoechst-labeled DNA; Orange, FITC-tubulin–labeled microtubules; green, H3K9me2. Scale bar, 10 μm. (**C**) Brightfield images of two-cell-stage embryos and blastocysts derived from WT (*n* = 55) and *Depdc7*^–/–^ (*n* = 50) female mice after IVF. Scale bar, 50 μm. (**D**) PCA plot of gene expression data obtained via transcriptome and proteome analysis from WT and *Depdc7*^–/–^ GV and MII oocytes. Each group includes 4 to 5 biological replicates, with ten oocytes per replicate. (**E**) Hoechst staining of chromosomes from four-cell embryos from WT and *Depdc7*^–/–^ mice. Scale bar, 10 μm. (**F**) Percentages of oocytes with the indicated ploidy are shown. 100% of WT oocytes (*n* = 19) were diploid, whereas 100% of *Depdc7*^–/–^ oocytes (*n* = 24) were triploid. *n* indicates the number of oocytes analyzed. All experiments were performed in at least three independent biological replicates. Source data are available online for this figure.

**Figure EV4:**
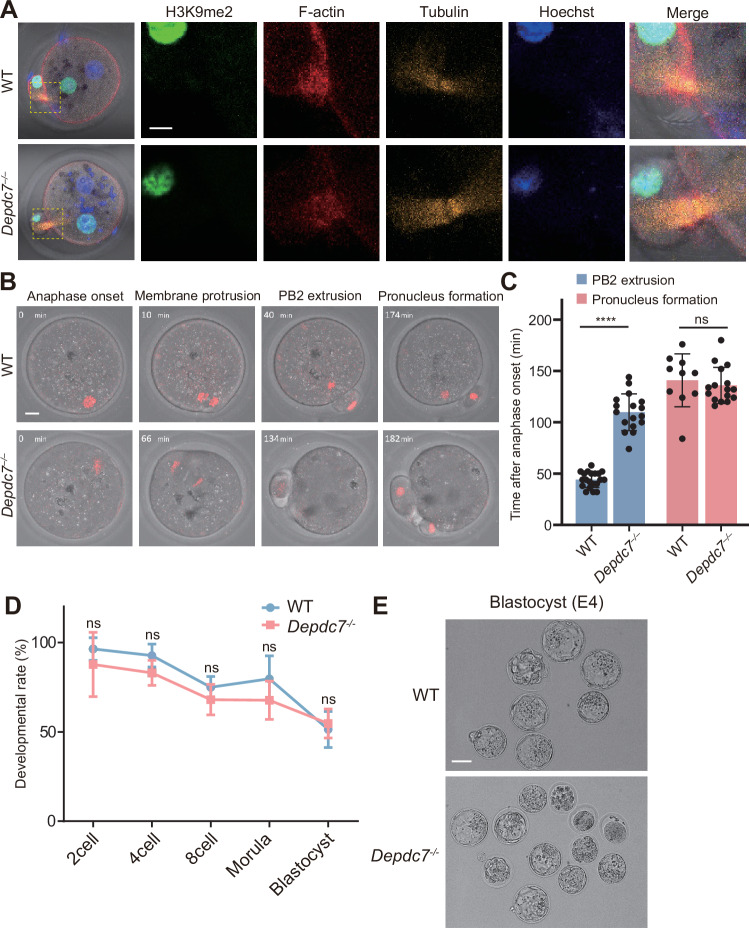
*Depdc7*^–/–^ oocytes can develop into blastocysts. (**A**) Immunofluorescence images of 2PN zygotes from WT and *Depdc7*^*–/–*^ mice after IVF. The polar body neck region is shown at higher magnification corresponding to Fig. 6B. Red, phalloidin-labeled F-actin; blue, Hoechst-labeled DNA; Orange, FITC-tubulin–labeled microtubules; green, H3K9me2. Scale bar, 5 μm. (**B**) Representative time-lapse images showing chromosome dynamics in WT and *Depdc7*^–/–^ oocyte following anaphase onset during parthenogenetic activation. Red, H2B-mScarlet-labeled chromosomes. Scale bar, 10 μm. (**C**) Quantification of the time required for PB2 extrusion and pronucleus (PN) formation in WT and *Depdc7*−/− oocytes. *n*  =  10–20 oocytes from three independent experiments per group. Data were presented as mean ± SD. ****p = 3.94*10^−12^, ^ns^*p* = 0.5980. Welch’s *t*-test. (**D**) The ratio of developmental stages between WT (*n* = 55) and *Depdc7*^–/–^ (*n* = 50) embryos after IVF. ^ns1^*p* = 0.4758, ^ns2^*p* = 0.1529, ^ns3^*p* = 0.3101, ^ns4^*p* = 0.2796, ^ns5^*p* = 0.6779, Student’s *t*-test. (**E**) Brightfield images of blastocysts obtained from WT and *Depdc7*^–/–^ mated female mice at 90 h after hCG injection. Scale bar, 50 μm. Source data are available online for this figure.

Despite the failure of cytokinesis in *Depdc7*^–/–^ oocytes, these oocytes could still be fertilized in vitro and form 2PN zygotes (Fig. 6A). To determine whether fertilization and second polar body extrusion occur normally in *Depdc7*^*–/–*^ oocytes, we collected 2PN zygotes following IVF. H3K9me2 staining confirmed that *Depdc7*^*–/–*^ oocytes were normally fertilized and formed one male and one female pronucleus (Fig. 6B). As shown in Fig. EV4A, a residual F-actin signal was detected at the polar body neck, whereas microtubules were no longer present at the intercellular bridge, indicating that the polar body had been fully separated. These results suggest that *Depdc7*^*–/–*^ oocytes can extrude a polar body after fertilization. We next performed live-cell imaging following parthenogenetic activation to monitor the process of polar body extrusion. Membrane protrusion occurred shortly after anaphase onset in WT oocytes, and the second polar body was extruded at ~40 min. In contrast, *Depdc7*^*–/–*^ oocytes failed to exhibit immediate membrane protrusion following anaphase onset, and second polar body extrusion occurred at approximately 110 min. Despite this difference, pronucleus formation occurred at a comparable time point in both groups, at approximately 140 min after anaphase onset (Fig. EV4B,C; Movies EV5 and EV6). Furthermore, the zygotes could develop normally to the blastocyst stage (Figs. 6C and EV4D). We then confirmed this result in vivo, and embryos were collected from the uteruses of WT and *Depdc7*^–/–^ mated female mice at 90 h after hCG injection. Similar results were obtained, and most embryos from *Depdc7*^–/–^ female mice could develop into blastocysts (Fig. EV4E). Thus, we speculated that the cytoplasmic maturation of *Depdc7*^–/–^ oocytes might be normal despite the interruption of nuclear maturation. To verify this hypothesis, we compared the transcriptomes and proteomes between WT and *Depdc7*^–/–^ oocytes and found that both the transcriptome and proteome of *Depdc7*^–/–^ oocytes were highly comparable with WT oocytes at different stages, as shown by the PCA plot (Fig. 6D; Dataset EV1). This suggested that the cytoplasmic maturation of the ovulated *Depdc7*^–/–^ oocytes was normal. However, although blastocyst embryos could develop, *Depdc7*^–/–^ females had no live births. Based on previous findings that ovulated *Depdc7*^–/–^ oocytes were tetraploid, we inferred that the embryos from *Depdc7*^–/–^ females would be multiploid. The embryonic karyotype was assessed by analyzing four-cell embryos, and the results showed that the embryos from *Depdc7*^–/–^ female mice were triploid while WT embryos were diploid (Fig. 6E,F), indicating that the infertility was likely due to the triploidy, which severely impaired normal embryonic development.

### Inhibiting CDK1 in *Depdc7*^*–/–*^ oocytes allows for the extrusion of PB1

**Figure 7:**
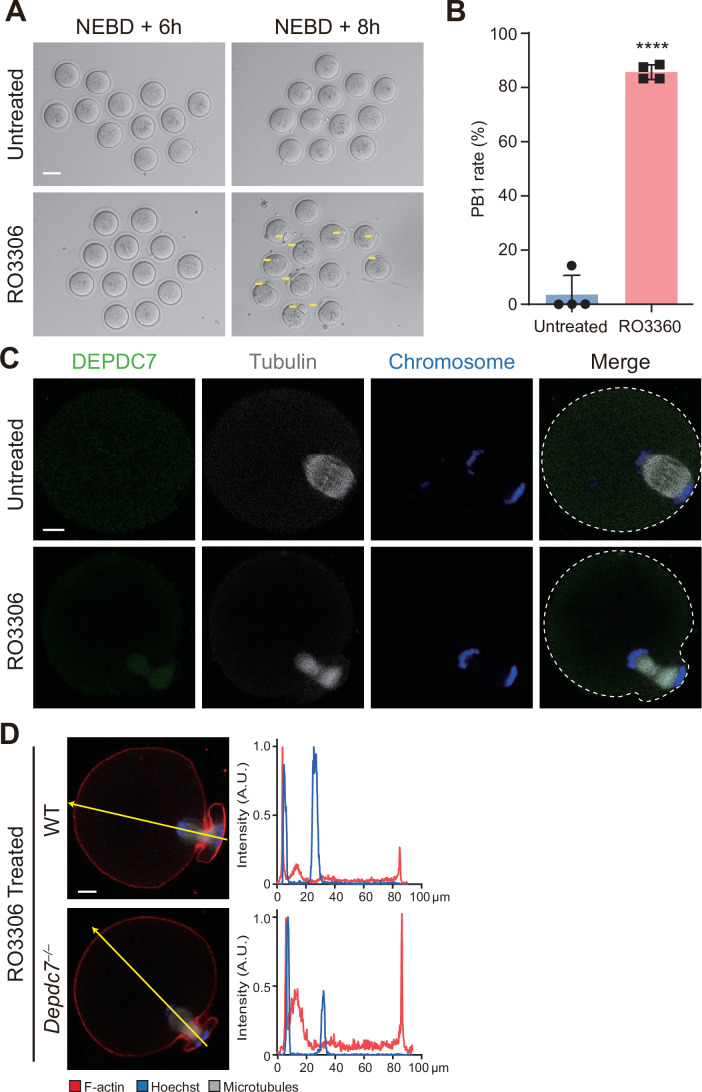
PB1 extrusion is rescued in *Depdc7*^*–/–*^ oocytes by RO3306. (**A**) Brightfield images of *Depdc7*^–/–^ oocytes treated with (*n* = 52) or without (*n* = 43) RO3306 starting at 6 h after NEBD. The yellow arrow points to PB1. Scale bar, 50 μm. (**B**) The PB1 extrusion rate in (**A**). Data were presented as mean ± SD. *P* = 2.63 × 10^−18^, Fisher’s exact test. (**C**) Immunofluorescence images of AI (anaphase I) in *Depdc7*^–/–^ oocytes treated with or without RO3306. Blue, Hoechst-labeled chromosomes; gray, FITC-tubulin–labeled microtubules; green, DEPDC7. Dashed lines indicate the oocyte boundary. Scale bar, 10 μm. (**D**) Immunofluorescence images of WT oocytes and *Depdc7*^–/–^ oocytes treated with RO3306. Red, phalloidin-labeled F-actin; gray, FITC-tubulin–labeled microtubules; blue, Hoechst-labeled DNA. Scale bar, 10 μm. The measurements of F-actin (red) and chromosomes (blue) intensity along the direction of the yellow arrows are shown at the right. *n* indicates the number of oocytes analyzed. All experiments were performed in at least three independent biological replicates. For intensity normalization in (**D**), the minimum intensity value along the line-scan was set to 0, the maximum value was set to 1.0, and all intermediate values were scaled accordingly. Source data are available online for this figure.

Although *Depdc7*^*–/–*^ oocytes failed to extrude the PB1, they were still able to extrude the PB2 upon fertilization (Figs. 2C and 6A). This observation suggested that PB1 extrusion depends on the actin cap, whereas PB2 extrusion does not. Previous studies have shown that cytoplasmic CDK1 activity plays a critical role in oocyte cytokinesis: inhibition of CDK1 alone is sufficient to drive oocyte division (Pomerantz et al, 2012; Wei et al, 2018). During normal meiosis, CDK1 activity transiently declines at the MI-to-MII transition and is rapidly reactivated at MII arrest, whereas CDK1 remains inactivated for a prolonged period following fertilization (Brunet and Maro, 2005; Viveiros and De La Fuente, 2019; Zhou and Homer, 2022). Based on these observations, we hypothesized that the actin cap may not be the decisive factor for PB2 extrusion. Instead, its primary function could be to facilitate timely spindle anchoring and membrane protrusion, particularly during meiosis I when CDK1 inactivation is transient. To explore this, we examined whether CDK1 inhibition during anaphase I could allow *Depdc7*^*–/–*^ oocytes to complete cytokinesis concurrently with homologous chromosome segregation. To test this hypothesis, we treated *Depdc7*^–/–^ oocytes with RO3306 (a CDK1 inhibitor) from 6 h post-NEBD, a suitable time that has been reported (Wei et al, 2018). As expected, most *Depdc7*^–/–^ oocytes could extrude PB1 2 h later (Fig. 7A,B), and the polar body contained chromosomes (Fig. 7C). We evaluated cortical actin fluorescence intensity in *Depdc7*^–/–^ oocytes treated with RO3306. Its intensity was markedly lower than that of a typical actin cap of a WT oocyte (Fig. 7D). These findings collectively suggested that the asymmetric extrusion of polar bodies in oocytes is determined by the balance of CDK1 temporal activity and cortical actin cap states.

## Discussion

The asymmetric subcortical positioning of the spindle and the establishment of cell polarity are crucial events for oocyte asymmetric division, and the actin cap is known to be important for oocyte polarity establishment and oocyte maturation. Here, we discovered the novel actin cap-localized protein DEPDC7, which bridges Ran-GTP signaling and the CDC42-NWASP-ARP2/3 complex pathway in regulating F-actin accumulation and actin cap formation. Oocytes lacking DEPDC7 cannot extrude PB1 due to the failure of actin cap formation, and this results in arrested tetraploid MII-like oocytes or triploid embryos after fertilization. Our study suggests a novel mechanism of DEPDC7-mediated actin cap establishment and oocyte maturation in oocytes.

**Figure 8:**
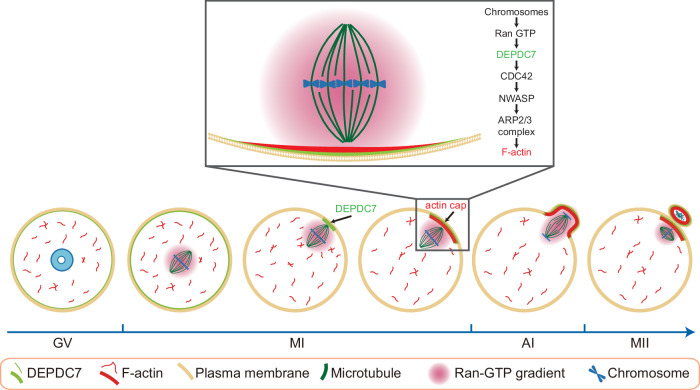
The proposed model for the process of oocyte actin cap formation. DEPDC7 shows weak expression beneath the cortex in GV-stage oocytes, and its expression gradually increases after NEBD. As the spindle migrates to the subcortex, DEPDC7 responds to the Ran-GTP signal, accumulating at the polarity region during MI. Next, proteins related to the actin cap signaling pathway are recruited and localized based on DEPDC7. Subsequently, F-actin accumulates within this zone, forming an actin cap. GV germinal vesicle, MI metaphase I, AI anaphase I, MII metaphase II.

Previous studies have established a signaling pathway for actin cap formation in which Ran-GTP is used as the orientation signal and the CDC42-NWASP-ARP2/3 complex is recruited to form the actin cap. However, the protein that transduces the signal from Ran-GTP to the actin cap has remained unknown. Another question is how F-actin accumulates in the cortex. It is generally considered that F-actin is anchored to the inside of the plasma membrane via p-ERM to maintain cortical tension (Fehon et al, 2010; Larson et al, 2010). However, the oocyte polarity region maintains dephosphorylated ERM when the chromosomes come close to the cortex. Therefore, nucleation of F-actin by the ARP2/3 complex may be localized to the cortex through some unknown proteins. In this study, we found that DEPDC7 is a key protein in polarized signal transduction and polarity establishment. Specifically, we found that DEPDC7 is located in the polar region and can regulate actin cap formation. The polar localization of DEPDC7 is regulated by Ran-GTP, and this in turn regulates the recruitment of related proteins to form the actin cap. Furthermore, DEPDC7 is a membrane-anchored protein that acts as a scaffold for F-actin accumulation. We propose that DEPDC7 responds to Ran-GTP signaling and becomes enriched at the plasma membrane adjacent to the chromosomes, where it may facilitate the recruitment of actin cap–associated proteins to the polarized cortex. The coordinated enrichment and assembly of these proteins ultimately give rise to actin cap formation (Fig. 8).

Previous studies have found that nuclear maturation is not synchronized with cytoplasmic maturation. Aneuploidy caused by nuclear maturation failure sometimes has little effect on fertilization and early embryonic development, but it often leads to post-implantation miscarriage. For example, the lack of CCNB3 (Li et al, 2019) or ELL3 (Zhu et al, 2025) leads to failure of nuclear maturation in oocytes, but these oocytes can still be fertilized and develop into blastocysts. Our study also confirms the asynchrony between nuclear and cytoplasmic maturation. Despite the failure to extrude PB1, *Depdc7*^–/–^ oocytes are still capable of fertilization and development into blastocysts. These results indicate that the cortical actin cap is essential for nuclear maturation but not for cytoplasmic maturation.

It should be noted that our results support an essential role of *Depdc7*^–/–^ in recruiting actin cap–associated proteins to the polarized cortical region, whereas the expression levels of these proteins appear unaffected (Fig. EV3C). Furthermore, the non-polar cortical F-actin was largely intact, as stable subcortical F-actin could still be observed in *Depdc7*^–/–^ oocytes. To assess cytoplasmic F-actin dynamics, we quantified GV-stage cytoplasmic F-actin intensity and found that it was largely unchanged in *Depdc7*^–/–^ oocytes (Appendix Fig. S2). Consistent with this, the spindle was still able to migrate toward the cortex during metaphase (Movies EV1–4). Collectively, these observations indicate that DEPDC7 plays a unique role in actin cap assembly, specifically affecting F-actin organization at the polarized cortex while largely sparing global F-actin dynamics.

It is well known that the actin cap is essential for polar body extrusion, and our study confirms that the actin cap is required for PB1 extrusion. However, some studies have shown that the inactivation of CDK1 is sufficient to enable oocyte cytokinesis (Pomerantz et al, 2012; Wei et al, 2018). Currently, little is known about the temporal coordination between CDK1 activity and cortical actin cap formation during oocyte cytokinesis. In this study, we found that while *Depdc7*^–/–^ oocytes could not extrude PB1, they were capable of extruding a polar body after fertilization despite the loss of cortical polarity. In addition, inhibiting CDK1 at the right time could promote PB1 extrusion in *Depdc7*^–/–^ oocytes. These results indicate that both CDK1 activity and the actin cap are essential for polar body extrusion. However, the actin cap is essential only for the extrusion of PB1, but not for PB2. As is well known, during the transition from meiosis I to meiosis II, CDK1 activity decreases transiently. CDK1 is rapidly reactivated to enter meiosis II and is maintained at a high level of activity at the MII stage. However, when oocytes are fertilized, CDK1 activity decreases and is maintained at a low level at the pronuclear stage (Brunet and Maro, 2005; Viveiros and De La Fuente, 2019; Zhou and Homer, 2022). Therefore, we speculate that the oocyte needs to prepare for this transition in advance by building up the actin cap in order to quickly extrude PB1 during AI in response to the temporary inactivation of CDK1. When the oocyte is fertilized, CDK1 is inactivated for a relatively long time to allow the oocyte to extrude PB2 independent of the actin cap. However, the mechanism through which the actin cap and CDK1 cooperate in oocyte PB1 extrusion requires further investigation. Furthermore, inhibition of CDK1 by RO3306 allowed *Depdc7*^*−/−*^ oocytes to extrude the PB1; this rescue was incomplete. The aneuploidy rate increased to ~40%, the fertilization rate was markedly reduced, and almost all embryos arrested before the four-cell stage (Appendix Fig. S3). The onset of anaphase during meiosis normally requires satisfaction of the spindle assembly checkpoint (SAC), ensuring that all kinetochores are properly attached to spindle microtubules. However, treatment with RO3306 prematurely triggered anaphase onset, thereby bypassing proper checkpoint control. Consequently, some erroneous kinetochore–microtubule attachments may persist and lead to an increased incidence of aneuploidy. Thus, premature progression into anaphase I induced by RO3306 treatment may reflect compromised cytoplasmic maturation of the oocyte, thereby increasing chromosome segregation errors and reducing the fertilization potential and developmental competence of the oocytes.

The study has some limitations. First, our Lat-A experiments could only demonstrate that cortical F-actin is not required for the maintenance of DEPDC7 polarity localization. But we could not directly determine whether F-actin is required for the initial formation of DEPDC7 polarity. Because the formation of the polarity domain depends on asymmetrically positioned chromosomes, which generate an asymmetric Ran-GTP gradient. However, chromosome asymmetry requires spindle migration to the cortex, a process driven by F-actin dynamics. Therefore, inhibiting F-actin disrupts spindle migration and prevents polarity establishment, making it difficult to distinguish a direct role of cortical F-actin in the initial formation of DEPDC7 enrichment. Whether F-actin is required for the initial establishment of the DEPDC7-enriched region remains to be determined. Second, although DEPDC7 is highly expressed in oocytes, its expression in other tissues appears to be relatively low. To date, functional studies of DEPDC7 remain very limited. Here, we report for the first time that DEPDC7 participates in regulating actin dynamics in oocytes. Whether DEPDC7 has similar functions in other cell types requires further investigation. Third, the precise molecular mechanism linking Ran-GTP signaling to DEPDC7 cortical localization and CDC42 recruitment remains unclear. We performed exploratory experiments, but did not obtain direct evidence of a physical interaction between DEPDC7 and Ran or CDC42. DEPDC7 may act downstream of Ran-GTP through indirect regulatory pathways or additional intermediates that remain to be identified. Further biochemical and mechanistic studies will be required to elucidate the molecular network connecting Ran-GTP signaling, DEPDC7, and CDC42-mediated actin remodeling during oocyte maturation.

To date, only a few studies have focused on the physiological role of *Depdc7*. Two studies reported that DEPDC7 acts as a tumor suppressor, and reduced expression of DEPDC7 in liver cancer cells promotes cancer progression (Liao et al, 2017a; Liao et al, 2017b). However, another study showed that knockdown of *Depdc7* inhibits cell proliferation and migration by affecting NF-κB activity (D’Andrea et al, 2014). In addition, three studies have shown that *Depdc7* may have potential associations with some diseases, including depression (Córdova-Palomera et al, 2015), developmental color agnosia (Nijboer et al, 2023), and sepsis (Thavarajah et al, 2020). However, all of the above studies lack an in-depth exploration of the physiological role of *Depdc7*. Here, we screened a gene expression profile and found that *Depdc7* has a unique expression profile pattern during oocyte maturation, and we revealed the specific physiological function of *Depdc7* during oocyte maturation and provided a new molecular mechanism for asymmetric division in oocytes.

In conclusion, we found that DEPDC7 acts as an early effector downstream of Ran-GTP and that it regulates F-actin accumulation and actin cap formation. DEPDC7 depletion interrupts PB1 extrusion by forming tetraploid oocytes with normal MII-like cytoplasmic maturation. These arrested tetraploid oocytes can be fertilized successfully, but they lead to triploid embryos, resulting in subsequent embryonic development failure and female infertility. We also identified the essential synergistic effect of CDK1 activity and actin cap formation for polar body extrusion during meiosis I.

## Methods

**Table:** Reagents and tools table

Reagent/resource	Reference or source	Identifier or catalog number
**Experimental models**		
C57BL6/J (*M. musculus*)	Charles River	N/A
C57BL6/J (*M. musculus*)	Cyagen	N/A
**Recombinant DNA**		
pcmv6-mDepdc7-HA	This study	N/A
pcmv6-mCdc42-HA	This study	N/A
pcmv6-mNwasp-HA	This study	N/A
pcmv6-mArp2-HA	This study	N/A
pcmv6-mRAN-HA	This study	N/A
Pcr3.1-H2B-mClover3	This study	N/A
**Antibodies**		
Rabbit anti-DEPDC7	Proteintech	26517-1-AP
Rabbit anti-ARP2	Proteintech	10922-1-AP
Rabbit anti-NWASP	Abclonal	A2576
Rabbit anti-CDC42	Proteintech	10155-1-AP
Rabbit anti-p-ERM	Proteintech	3726S
Human anti-CREST	Immunovision	HCT-0100
Rabbit anti-H3K9me2	Proteintech	86990-2-RR
FITC-tubulin	Thermo Fisher	CL488-66240
Alexa Fluor 647 Donkey anti-Rabbit IgG	Thermo Fisher	A32795
Alexa Fluor 488 Donkey anti-Rabbit IgG	Thermo Fisher	A21206
Alexa Fluor™ Plus 488 Goat anti-Human IgG (H + L) Cross-Adsorbed Secondary Antibody	Invitrogen	A48276
Rabbit anti-VINCULIN	CST	13901
Anti-Rabbit IgG HRP	Abmart	M21002L
**Oligonucleotides and other sequence-based reagents**		
Depdc7-RT-F: CAAGTCGGACAGTTTGGAGGAC	This study	N/A
Depdc7 -RT-R: GTTGTAGCAGCCGTCCAATCGT	This study	N/A
Gapdh-RT-F: CATCACTGCCACCCAGAAGACTG	This study	N/A
Gapdh-RT-R: ATGCCAGTGAGCTTCCCGTTCAG	This study	N/A
Depdc7-genotype-F1: CTTCCTGGTTTTACTCAAGTCCCTA	This study	N/A
Depdc7- genotype-R1: CCATTGCAGAAGCAGAACTTAACA	This study	N/A
Depdc7- genotype-F2: CCTCCAGCTGTCAGTATGAATGTA	This study	N/A
Depdc7- genotype-R2: CCATTGCAGAAGCAGAACTTAACA	This study	N/A
**Chemicals, Enzymes and other reagents**		
Alexa FLUOR 532 PHALLO	Thermo Fisher	A22282
Hoechst 33342	MedChemExpress	HY-15559
M2 medium	Aibei Biotechnology	M1205
Milrinone	MCE	HY-14252
Hyaluronidase	Aibei Biotechnology	M2215
Fetal bovine serum (FBS)	Gibco	A5256701
Bovine serum albumin (BSA)	Sigma	B2064
Hoechst	Thermo Fisher Scientific	R37605
Paraformaldehyde	Sigma	FX0415
ECL Western Blotting Substrate	Tanon	180-5001
RO3306	MedChemExpress	HY-12529
LAT-A	MedChemExpress	HY-16929
Monastrol	MedChemExpress	HY-101071A
**Software**		
GraphPad Prism	GraphPad Software Inc.	Version 9.4
ImageJ (Fiji)	github.com/fiji/fiji	
Adobe Illustrator	Adobe	Version 2024
ZEISS Zen Lite	ZEISS	
**Other**		
RNeasy Plus Micro Kit	QIAGEN	74034
PrimeScript RT reagent Kit with gDNA Eraser	Takara	RR047A
TB Green Premix Ex Taq	TaKara	RR820A
KOD-Plus Mutagenesis Kit	TOYOBO	SMK-101
HiScribe T7 ARCA mRNA Kit	New England Biolabs	E2060S
Zeiss LSM 880	ZEISS	N/A
Chemiluminescent imaging system	Tanon	5200

### Generation of *Depdc7*^*–/–*^ mice

The *Depdc7*^*–/–*^ mouse model was generated in collaboration with Cyagen Biosciences. Briefly, exons 2–4 of *Depdc7* were deleted using the CRISPR/Cas9 system. Cas9 and sgRNA were injected into the fertilized eggs of C57BL/6J mice, and positive F0 mice were identified by PCR and sequencing analysis. The stable inheritable heterozygous F1 mouse was obtained by mating F0 mice with C57BL/6 J mice. Homozygous *Depdc7*^*–/–*^ mice were obtained by crossing heterozygous mice. All mice were maintained under specific pathogen-free (SPF) conditions in a temperature- and humidity-controlled animal facility with a 12 h light/dark cycle and ad libitum access to food and water. The primers used for mouse genotyping are listed in the Reagents and Tools Table. All mouse experiments were reviewed and approved by the Ethics Committee of the Shanghai Medical College of Fudan University (Approval number 2023-EKYY-59JZS).

For female fertility assay, each 6-8 weeks *Depdc7*^*–/–*^ female mouse was caged with one 6–8 weeks WT female mouse and one 8–10 weeks male mouse, the number of offspring was recorded. To accurately assess whether the *Depdc7*^*–/–*^ female mouse had mated, vaginal plug checks were performed during the first two months after pairing to ensure that each female had successfully copulated. For the male fertility assay, each 8–10 weeks male mouse was caged with two 6–8 weeks WT female mice, and the number of offspring was recorded.

### Oocyte and embryo collection and in vitro culture

To obtain oocytes, mice were intraperitoneally injected with 10 IU of pregnant mare serum gonadotropin (PMSG). After 47 h, GV oocytes were obtained from the ovary and cultured in G2 medium containing 2.5 µM milrinone at 37 °C with 5% CO₂ until further use. To get MI/MII oocytes, mice were injected with 10 IU of human chorionic gonadotropin (hCG) at 47 h after PMSG injection. Metaphase I (MI) oocytes were isolated from ovaries by puncturing the antral follicles with a fine needle on the stage of a dissecting microscope at 8 h after hCG injection. MII oocytes were isolated from the oviducts 14 h after hCG injection. Oocyte-cumulus complexes were transferred to M2 medium containing hyaluronidase to remove cumulus cells, and the oocytes were washed and cultured in M2 medium at 37 °C covered by mineral oil.

To obtain embryos in vivo, super-ovulated female mice were mated with male mice after hCG injection. The mated female mice were examined the following morning for the presence of vaginal plugs and then separated from the males. Four cell-stage embryos were collected from the oviducts of mated females 60 h after hCG injection, and blastocyst-stage embryos were collected from the uteruses of the mated females 90 h after hCG injection.

For in vitro fertilization (IVF) and embryo culture, HTF and KSOM media were pre-warmed at 37 °C and 5% CO_2_. Oocyte-cumulus complexes were transferred into HTF and cultured with capacitated sperm, and 5 h later, 2PN zygotes were washed of excess sperm and transferred into KSOM medium for further embryo culturing. Developmental rates of embryos were calculated based on the number of normally fertilized eggs (2PN). It was defined as the proportion of 2PN embryos that reached the given cleavage stage. Data were presented as mean ± SD.

### Plasmid construction and mRNA synthesis

Full-length mouse *Depdc7*, *Cdc42*, and *Ran* transcripts were amplified from mouse GV oocyte cDNA, and full-length mouse *Nwasp* and *Arp2* were synthesized by the iGene Biotechnology company. All cDNAs were cloned into the PCMV6 entry vector with an HA tag at the C-terminus. H3 deletion mutants of *Depdc7* and *Ran-T24N* mutants were created with the KOD-Plus Mutagenesis Kit according to the manufacturer’s instructions (SMK-101, TOYOBO), and clones were confirmed by Sanger sequencing. The mRNA was transcribed in vitro from purified linear double-stranded DNA templates, and the HiScribe T7 ARCA mRNA Kit (with tailing) (New England Biolabs) was used for the in vitro transcription reaction and purification.

### Immunofluorescence microscopy

Oocytes were fixed with 2% paraformaldehyde and 0.05% Triton X-100 in PBS for 30 min and permeabilized in PBS containing 0.5% Triton X-100 for 1 h at room temperature. The oocytes were then incubated in a blocking solution (PBS, 3% BSA, 0.3% Triton X-100, and 0.1% Tween-20) at 37 °C for 1 h. All primary antibodies were incubated with the oocytes in a blocking buffer at 4 °C overnight or at 37 °C for 2 h, and secondary antibodies were incubated with the oocytes at 37 °C for 1 h. The primary antibodies were anti-DEPDC7 (1:200 dilution, Proteintech), anti-ARP2 (1:100 dilution, Proteintech), anti-NWASP (1:50 dilution, Abclonal), anti-CDC42 (1:50 dilution, Proteintech), anti-p-ERM (1:200 dilution, Proteintech), and FITC-anti-tubulin (1:100 dilution, Thermo). The secondary antibodies were Alexa Fluor 647-conjugated anti-rabbit IgG (1:200 dilution, CST), Alexa Fluor 647-conjugated anti-mouse IgG (1:200 dilution, CST), and Alexa Fluor 488-conjugated anti-rabbit IgG (1:400 dilution, Invitrogen). F-actin was labeled with phalloidin (1:400 dilution, Thermo) at 4 °C overnight. The chromosomes were labeled with Hoechst 33342 (20 μg/ml, HY-15559, MedChemExpress) before imaging. The samples were maintained in PBS and imaged with a confocal laser-scanning microscope (ZEISS LSM 880).

### Fluorescent intensity measurement

The direction of the line-scan intensity measurements is shown in the figures. The fluorescence intensity was quantified using ZEN software (Zeiss) and exported into Microsoft Excel for further analysis. For normalization, the minimum intensity value along the line-scan was set to 0 and the maximum value was set to 1.0, and all intermediate values were scaled accordingly. Specifically, for normalization in Fig. 1C, the mean fluorescence intensity along the entire line-scan was set to 1, and the fluorescence intensity at each point was normalized to this value.

### Microinjection of mouse oocytes

Microinjection was performed using a Leica Hoffman microscope (DMi8, Leica) equipped with a TransferMan 4r micromanipulator, InjectMan 4, and FemtoJet 4i (Eppendorf). Oocytes were microinjected with 3–5 pl of mRNA. After injection, the oocytes were released into fresh medium and further cultured at 37 °C and 5% CO_2_. Oocytes were collected for subsequent analyses at the indicated time points.

### Live-cell imaging

GV oocytes were allowed to overexpress H2B-mScarlet for 2 h and were then released in order to monitor chromosome dynamics during oocyte maturation. An LSM 880 confocal laser-scanning microscope (Zeiss) was used for live-cell imaging with the following parameters: environmental chamber: 37 °C, time-lapse: 30-min intervals for 14 h, 40×/1.1 NAC-Apochromat lens, 3 μm per layer scans of the entire oocyte.

### Single-cell RNA sequencing from oocytes

Each sample included five oocytes, and RNA-seq libraries were prepared using the cDNA Synthesis, Amplification, and Library Generation Kit (E6420, NEB). RNA quality was determined by gel electrophoresis and with Qubit (Thermo). RNA samples were sequenced on an Illumina Novaseq 6000 instrument by Genergy Biotechnology Co. Ltd. The raw data were processed by trim galore software, and data quality was checked by FastQC v0.11.2. The read length was 2 × 150 bp. Clean reads were aligned to the mouse genome mm10 using STAR and StringTie. The expression levels of the genes were quantified using normalized fragments per kilobase million (FPKM).

### Single-cell LC-MS/MS analysis

Collected oocytes were processed for ultrasensitive proteomics using the Ex Ultra-Low Sample Preparation Kit (OSFP0004, Shanghai Omicsolution Co., Ltd., China) following the manufacturer’s instructions. Briefly, oocytes underwent protein extraction, denaturation, reduction, alkylation, and enzymatic digestion, followed by desalting of peptides. Cleaned peptides were dried under vacuum and resuspended in 0.1% formic acid prior to LC-MS/MS analysis. Peptide separation was performed on a Vanquish Neo UHPLC system (Thermo Fisher Scientific, MA, USA) coupled to a timsTOF HT mass spectrometer (Bruker Daltonik, Bremen, Germany). Samples were separated using a 25 min gradient on an AUR3-5075 C18 column (15 cm × 75 μm i.d., 1.7 μm particle size; IonOpticks) and analyzed in dia-PASEF mode. Raw MS data were processed using Spectronaut19 with BGS Factory default settings to obtain protein identification and quantification results.

### Drug treatment

For acute actin cap disruption, MII oocytes were treated with 10 µM Latrunculin A (LAT-A, Thermo, 2 mM stock) or incubated in DMSO for 20 min and then fixed for immunofluorescence assays. RO3306 (Sigma-Aldrich; 20 mM stock) was used to inhibit CDK1 activity at a concentration of 10 μM. *Depdc7*^*–/–*^ oocytes were incubated in culture medium with DMSO or RO3306 for 6 h after nuclear envelope breakdown (NEBD). Monastrol (100 μM, HY-101071A, MCE) was added to the M2 medium of MII oocytes for 1.5 h for chromosome spreads.

### Chromosome spreads

Four-cell embryos were briefly exposed to acid Tyrode’s solution to remove the zona pellucida under a microscope. After a brief recovery in M2 medium, the embryos were exposed to 0.5% citric acid to expand their volume. For fixation, the zona pellucida-free embryos were transferred to a drop of chromosome spread solution (0.15% Triton X-100 and 3 mM dithiothreitol in 1% paraformaldehyde in distilled water (pH 9.2)) on a microscope slide. The dried chromosome spreads were stained with Hoechst for DNA counterstaining, and the images were captured using a confocal laser-scanning microscope (ZEISS LSM 880).

### Statistical analysis

Principal component analysis (PCA) of the RNAseq and proteomics data was performed using the FactoMineR (version 2.11) R package and visualized with the factoextra (version 1.0.7) R package. Comparisons of categorical variables were performed using chi-square tests, and Fisher’s exact test was used to compare the significance of paired samples. Statistically significant values were *P < 0.05, **P < 0.01, ***P < 0.001, and ****P < 0.0001. All statistical analyses were performed using R version 4.1.0, and all graphical outputs were generated by GraphPad Prism. Oocytes showing degeneration, fragmentation, or abnormal morphology before imaging or microinjection were excluded based on preestablished criteria. No other samples or data points were excluded from the analysis. The experiments were not randomized, and the investigators were not blinded to allocation during the experiments and outcome assessment.

## Supplementary information

Appendix

Peer Review File

Dataset EV1

Movie EV1

Movie EV2

Movie EV3

Movie EV4

Movie EV5

Movie EV6

Source data Fig. 1

Source data Fig. 2

Source data Fig. 3

Source data Fig. 4

Source data Fig. 5

Source data Fig. 6

Source data Fig. 7

EV Figures Source Data

Expanded View Figures

## Data Availability

Raw RNA sequencing data generated in this study have been deposited in the Genome Sequence Archive (GSA) database (no. CRA042768: https://ngdc.cncb.ac.cn/gsa/search?searchTerm=CRA042768) The source data of this paper are collected in the following database record: biostudies:S-SCDT-10_1038-S44319-026-00842-0.

## References

[CR1] Azoury J, Lee KW, Georget V, Hikal P, Verlhac MH (2011) Symmetry breaking in mouse oocytes requires transient F-actin meshwork destabilization. Development 138:2903–2908 21653611 10.1242/dev.060269

[CR2] Azoury J, Lee KW, Georget V, Rassinier P, Leader B, Verlhac MH (2008) Spindle positioning in mouse oocytes relies on a dynamic meshwork of actin filaments. Curr Biol 18:1514–1519 18848445 10.1016/j.cub.2008.08.044

[CR3] Brunet S, Maro B (2005) Cytoskeleton and cell cycle control during meiotic maturation of the mouse oocyte: integrating time and space. Reproduction 130:801–811 16322540 10.1530/rep.1.00364

[CR4] Consonni SV, Maurice MM, Bos JL (2014) DEP domains: structurally similar but functionally different. Nat Rev Mol Cell Biol 15:357–362 24739740 10.1038/nrm3791

[CR5] Córdova-Palomera A, Fatjó-Vilas M, Palma-Gudiel H, Blasco-Fontecilla H, Kebir O, Fañanás L (2015) Further evidence of DEPDC7 DNA hypomethylation in depression: a study in adult twins. Eur Psychiatry 30:715–718 25952135 10.1016/j.eurpsy.2015.04.001

[CR6] D’Andrea EL, Ferravante A, Scudiero I, Zotti T, Reale C, Pizzulo M, De La Motte LR, De Maio C, Mazzone P, Telesio G et al (2014) The dishevelled, EGL-10 and pleckstrin (DEP) domain-containing protein DEPDC7 binds to CARMA2 and CARMA3 proteins, and regulates NF-κB activation. PLoS ONE 9:e116062 25541973 10.1371/journal.pone.0116062 PMC4277425

[CR7] Dehapiot B, Carrière V, Carroll J, Halet G (2013) Polarized Cdc42 activation promotes polar body protrusion and asymmetric division in mouse oocytes. Dev Biol 377:202–212 23384564 10.1016/j.ydbio.2013.01.029 PMC3690527

[CR8] Dehapiot B, Halet G (2013) Ran GTPase promotes oocyte polarization by regulating ERM (Ezrin/Radixin/Moesin) inactivation. Cell Cycle 12:1672–1678 23656777 10.4161/cc.24901 PMC3713125

[CR9] Deng M, Suraneni P, Schultz RM, Li R (2007) The Ran GTPase mediates chromatin signaling to control cortical polarity during polar body extrusion in mouse oocytes. Dev Cell 12:301–308 17276346 10.1016/j.devcel.2006.11.008

[CR10] Dumont J, Million K, Sunderland K, Rassinier P, Lim H, Leader B, Verlhac M-H (2007) Formin-2 is required for spindle migration and for the late steps of cytokinesis in mouse oocytes. Dev Biol 301:254–265 16989804 10.1016/j.ydbio.2006.08.044

[CR11] Fabritius AS, Ellefson ML, McNally FJ (2011) Nuclear and spindle positioning during oocyte meiosis. Curr Opin Cell Biol 23:78–84 20708397 10.1016/j.ceb.2010.07.008 PMC2994957

[CR12] Falginella FL, Kravec M, Drabinová M, Paclíková P, Bryja V, Vácha R (2022) Binding of DEP domain to phospholipid membranes: more than just electrostatics. Biochim Biophys Acta Biomembr 1864:183983 35750206 10.1016/j.bbamem.2022.183983

[CR13] Fehon RG, McClatchey AI, Bretscher A (2010) Organizing the cell cortex: the role of ERM proteins. Nat Rev Mol Cell Biol 11:276–287 20308985 10.1038/nrm2866 PMC2871950

[CR14] Larson SM, Lee HJ, Hung PH, Matthews LM, Robinson DN, Evans JP (2010) Cortical mechanics and meiosis II completion in mammalian oocytes are mediated by myosin-II and Ezrin-Radixin-Moesin (ERM) proteins. Mol Biol Cell 21:3182–3192 20660156 10.1091/mbc.E10-01-0066 PMC2938384

[CR15] Li Y, Wang L, Zhang L, He Z, Feng G, Sun H, Wang J, Li Z, Liu C, Han J et al (2019) Cyclin B3 is required for metaphase to anaphase transition in oocyte meiosis I. J Cell Biol 218:1553–1563 30770433 10.1083/jcb.201808088 PMC6504906

[CR16] Liao Z, Wang X, Lin D, Zou Q (2017a) Construction and identification of the RNAi recombinant lentiviral vector targeting human DEPDC7 gene. Interdiscip Sci Comput Life Sci 9:350–356 10.1007/s12539-016-0162-y 27016254

[CR17] Liao Z, Wang X, Wang X, Li L, Lin D (2017b) DEPDC7 inhibits cell proliferation, migration and invasion in hepatoma cells. Oncol Lett 14:7332–7338 29344171 10.3892/ol.2017.7128 PMC5755016

[CR18] Maro B, Johnson MH, Webb M, Flach G (1986) Mechanism of polar body formation in the mouse oocyte: an interaction between the chromosomes, the cytoskeleton and the plasma membrane. Development 92:11–32 3723057

[CR19] Namgoong S, Kim NH (2016) Roles of actin binding proteins in mammalian oocyte maturation and beyond. Cell Cycle 15:1830–1843 27152960 10.1080/15384101.2016.1181239 PMC4968894

[CR20] Nijboer TCW, Hessel EVS, van Haaften GW, van Zandvoort MJ, van der Spek PJ, Troelstra C, de Kovel CGF, Koeleman BPC, van der Zwaag B, Brilstra EH et al (2023) Identification of candidate genes for developmental colour agnosia in a single unique family. PLoS ONE 18:e0290013 37672513 10.1371/journal.pone.0290013 PMC10482254

[CR21] Pomerantz Y, Elbaz J, Ben-Eliezer I, Reizel Y, David Y, Galiani D, Nevo N, Navon A, Dekel N (2012) From ubiquitin-proteasomal degradation to CDK1 inactivation: requirements for the first polar body extrusion in mouse oocytes. FASEB J 26:4495–4505 22859367 10.1096/fj.12-209866

[CR22] Schuh M, Ellenberg J (2008) A new model for asymmetric spindle positioning in mouse oocytes. Curr Biol 18:1986–1992 19062278 10.1016/j.cub.2008.11.022

[CR23] Thavarajah T, Dos Santos CC, Slutsky AS, Marshall JC, Bowden P, Romaschin A, Marshall JG (2020) The plasma peptides of sepsis. Clin Proteomics 17:26 32636717 10.1186/s12014-020-09288-5 PMC7331219

[CR24] Verlhac MH, Lefebvre C, Guillaud P, Rassinier P, Maro B (2000) Asymmetric division in mouse oocytes: with or without Mos. Curr Biol 10:1303–1306 11069114 10.1016/s0960-9822(00)00753-3

[CR25] Viveiros MM, De La Fuente R (2019) Regulation of mammalian oocyte maturation. In: Leung PCK, Adashi EY (eds) The ovary, 3rd edn. Academic Press, pp 165–180

[CR26] Wang H, Li Y, Yang J, Duan X, Kalab P, Sun SX, Li R (2020) Symmetry breaking in hydrodynamic forces drives meiotic spindle rotation in mammalian oocytes. Sci Adv 6:eaaz5004 32284983 10.1126/sciadv.aaz5004 PMC7124937

[CR27] Wei Z, Greaney J, Zhou C, A. Homer H (2018) Cdk1 inactivation induces post-anaphase-onset spindle migration and membrane protrusion required for extreme asymmetry in mouse oocytes. Nat Commun 9:4029 30279413 10.1038/s41467-018-06510-9 PMC6168559

[CR28] Yi K, Rubinstein B, Unruh JR, Guo F, Slaughter BD, Li R (2013) Sequential actin-based pushing forces drive meiosis I chromosome migration and symmetry breaking in oocytes. J Cell Biol 200:567–576 23439682 10.1083/jcb.201211068 PMC3587830

[CR29] Yi K, Unruh JR, Deng M, Slaughter BD, Rubinstein B, Li R (2011) Dynamic maintenance of asymmetric meiotic spindle position through Arp2/3-complex-driven cytoplasmic streaming in mouse oocytes. Nat Cell Biol 13:1252–1258 21874009 10.1038/ncb2320 PMC3523671

[CR30] Zhou C, Homer HA (2022) The oocyte spindle midzone pauses Cdk1 inactivation during fertilization to enable male pronuclear formation and embryo development. Cell Rep 39:110789 35508138 10.1016/j.celrep.2022.110789

[CR31] Zhu S, Xie P, Yang Y, Wang Y, Zhang C, Zhang Y, Si S, Zhang J, He J, Si H et al (2025) Maternal ELL3 loss-of-function leads to oocyte aneuploidy and early miscarriage. Nat Struct Mol Biol 32:381–392 39820605 10.1038/s41594-024-01471-8

[CR32] Zhu Z-Y, Chen D-Y, Li J-S, Lian L, Lei L, Han Z-M, Sun Q-Y (2003) Rotation of meiotic spindle is controlled by microfilaments in mouse oocytes1. Biol Reprod 68:943–946 12604646 10.1095/biolreprod.102.009910

