# Supplementary material for: The mechanisms of DEPDC7-mediated actin cap establishment and meiosis progression in oocytes
Source: EMBO Rep. 2026 Jun 22;27(15):4412–32. doi: 10.1038/s44319-026-00842-0 (PMC13457618; doi:10.1038/s44319-026-00842-0)
Supplement: Supplementary file 1 [file 44319_2026_842_MOESM18_ESM.pdf]

Expanded View Figures

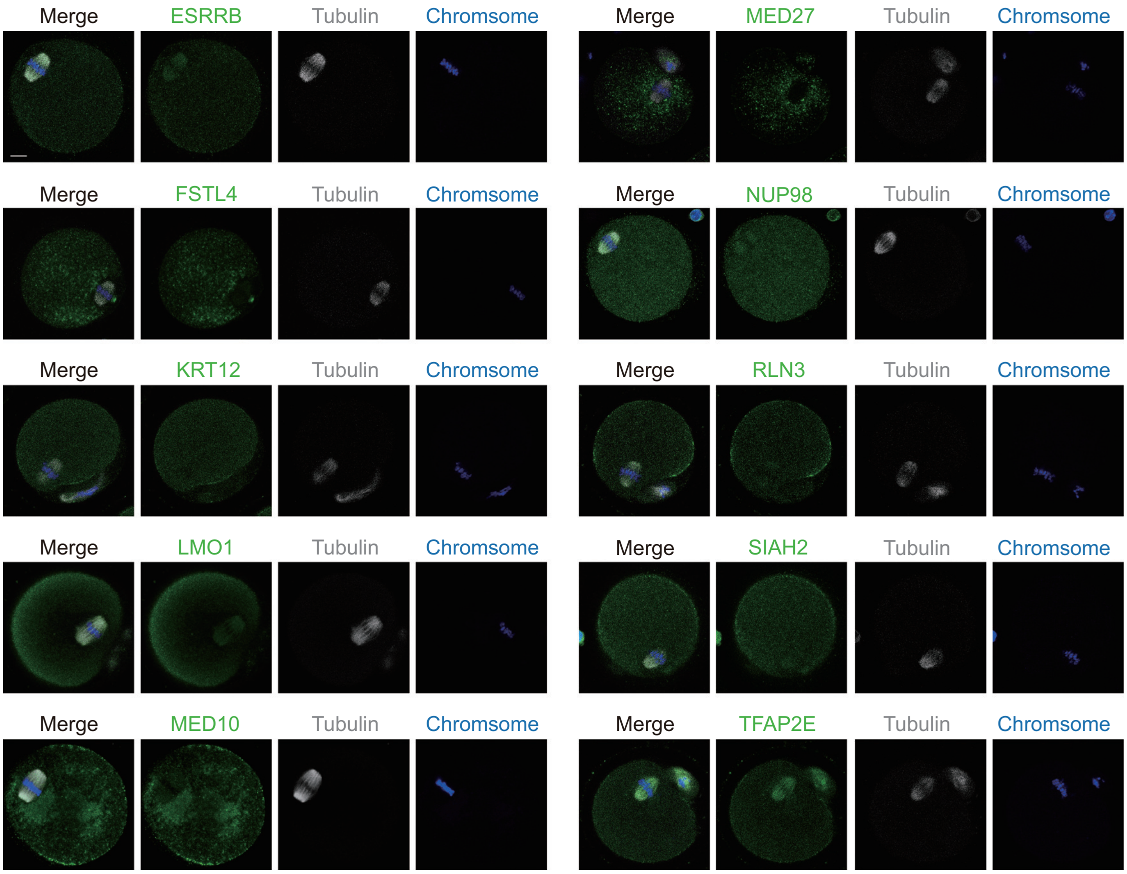

Figure EV1. Immunofluorescence staining of the indicated protein in oocytes.

The protein shows high expression in the oocyte. blue, Hoechst-labeled chromosome; gray, FITC-tubulin-labeled microtubules; green, indicated proteins. Scale bar, 10 μm. Source data are available online for this figure.

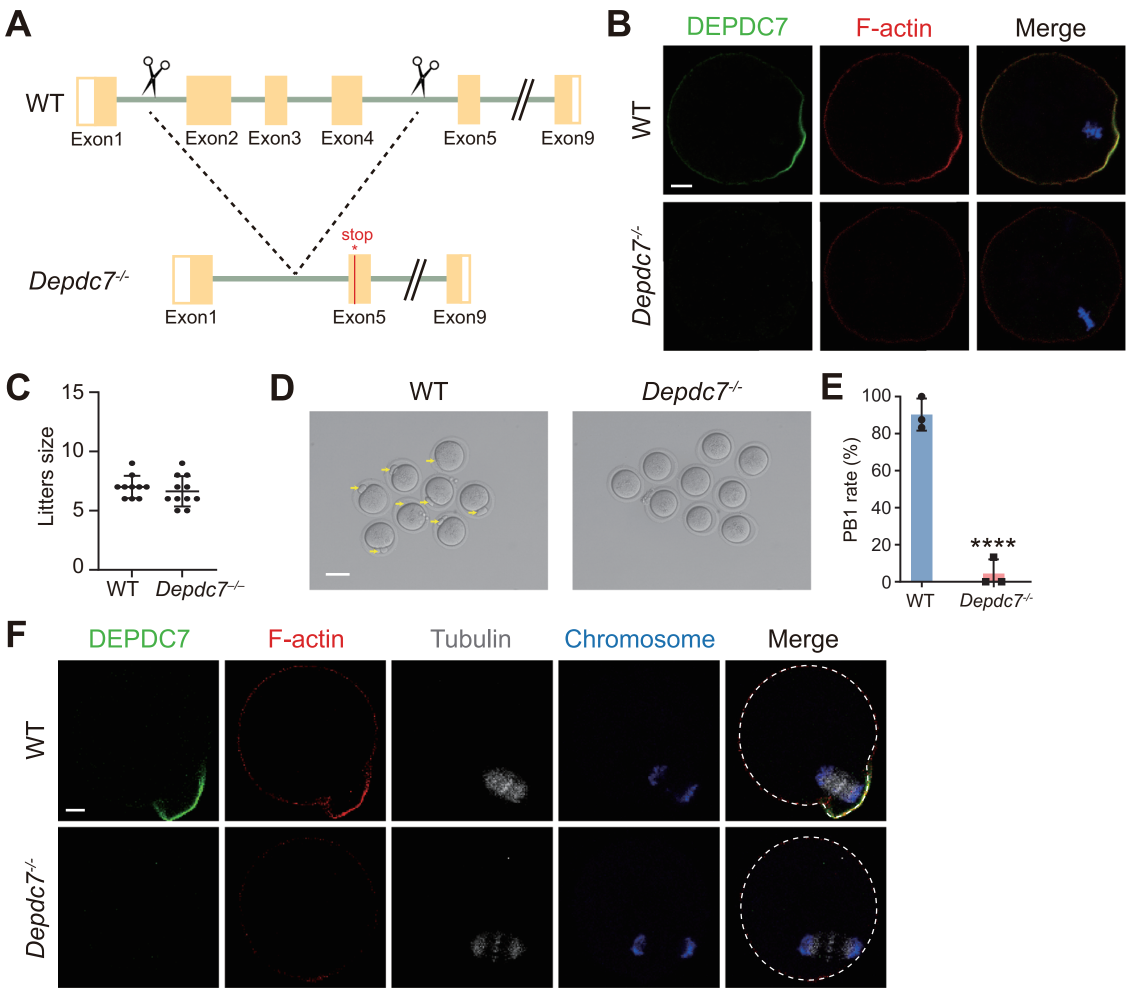

Figure EV2. Oocyte from *Depdc7*$^{-/-}$ mice could not extrude PB1.

(**A**) Schematic diagram of the genome of the *Depdc7* knockout mouse. Exons 2–4 were knocked out, leading to premature termination in exon 5. (**B**) Immunofluorescence staining of ovulated oocytes from WT ($n = 35$) and *Depdc7*$^{-/-}$ ($n = 29$) mice. Red, phalloidin-labeled F-actin; blue, Hoechst-labeled DNA; green, DEPDC7. Scale bar, 10 µm. (**C**) Fertility assay of WT ($n = 10$) and *Depdc7*$^{-/-}$ ($n = 11$) male mice. Data were presented as mean ± SD. (**D**) Brightfield image of WT ($n = 40$) or *Depdc7*$^{-/-}$ ($n = 30$) oocytes during in vitro maturation. The yellow arrow points to PB1. Scale bar, 50 µm. (**E**) The PB1 extrusion rate in (**D**). Data were presented as mean ± SD. $P = 2.05 \times 10^{-9}$, Fisher's exact test. (**F**) Immunofluorescence images of AI (anaphase I) in WT ($n = 15$) and *Depdc7*$^{-/-}$ ($n = 13$) oocytes. Red, phalloidin-labeled F-actin; blue, Hoechst-labeled chromosome; gray, FITC-tubulin-labeled microtubules; green, DEPDC7. Dashed lines indicate the oocyte boundary. Scale bar, 10 µm. *n* indicates the number of oocytes analyzed. All experiments were performed in at least three independent biological replicates. Source data are available online for this figure.

 © The Author(s)

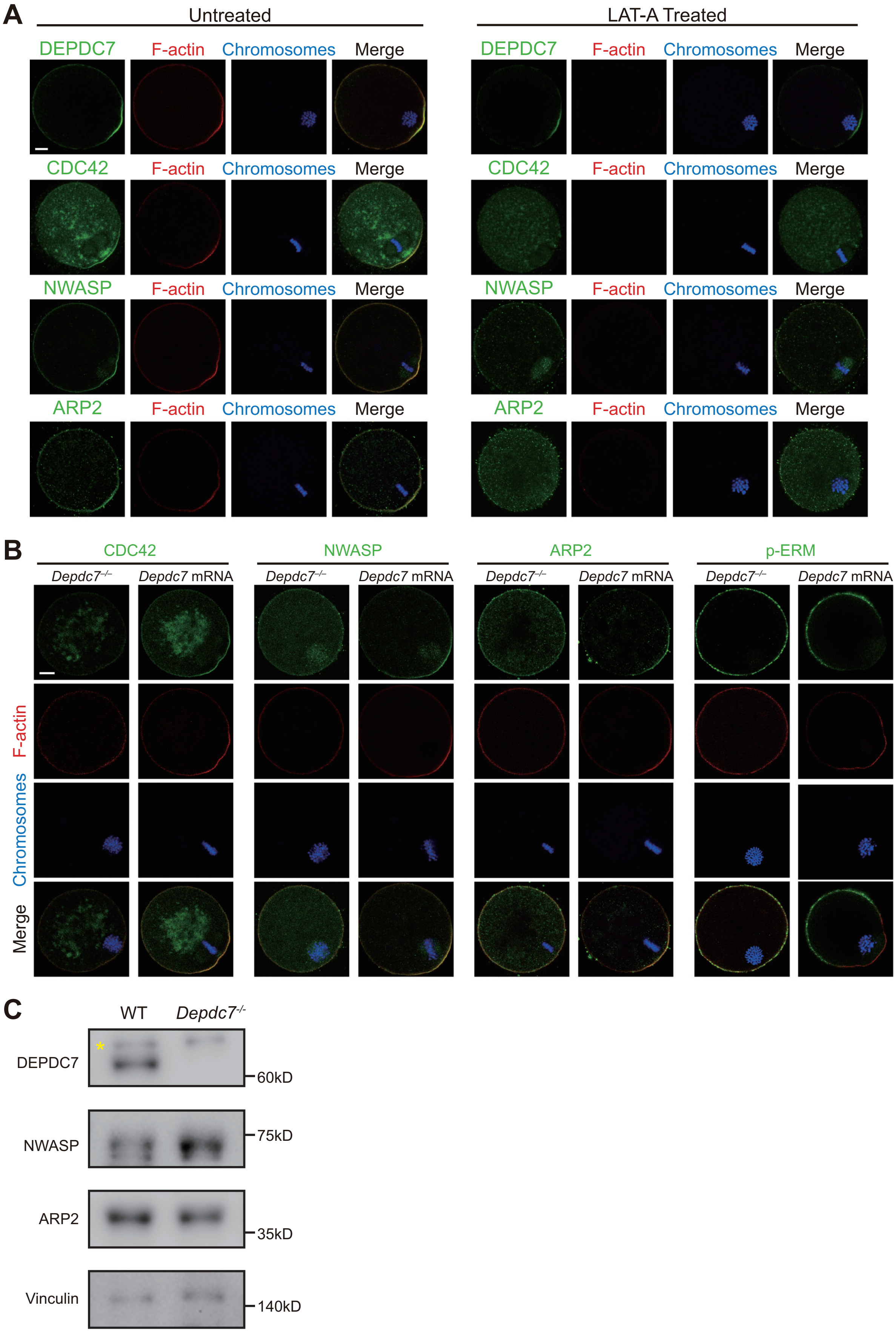
A
Untreated
LAT-A Treated
DEPDC7
F-actin
Chromosomes
Merge
CDC42
NWASP
ARP2
B
CDC42
NWASP
ARP2
p-ERM
Depdc7−/−
Depdc7 mRNA
F-actin
Chromosomes
Merge
C
WT
Depdc7−/−
DEPDC7
*
60kD
NWASP
75kD
ARP2
35kD
Vinculin
140kD

Figure EV3. Depletion of DEPDC7 does not affect the expression of related proteins.

(**A**, **B**) Full-channel images corresponding to Fig. 4B, C. (**C**) Immunoblot of DEPDC7, NWASP, ARP2, and vinculin in MII oocytes from WT and *Depdc7*$^{-/-}$ mice. 60 oocytes were collected from each group for immunoblotting, and the results were repeated three times. The yellow star indicates an unspecific band. Source data are available online for this figure.

© The Author(s)

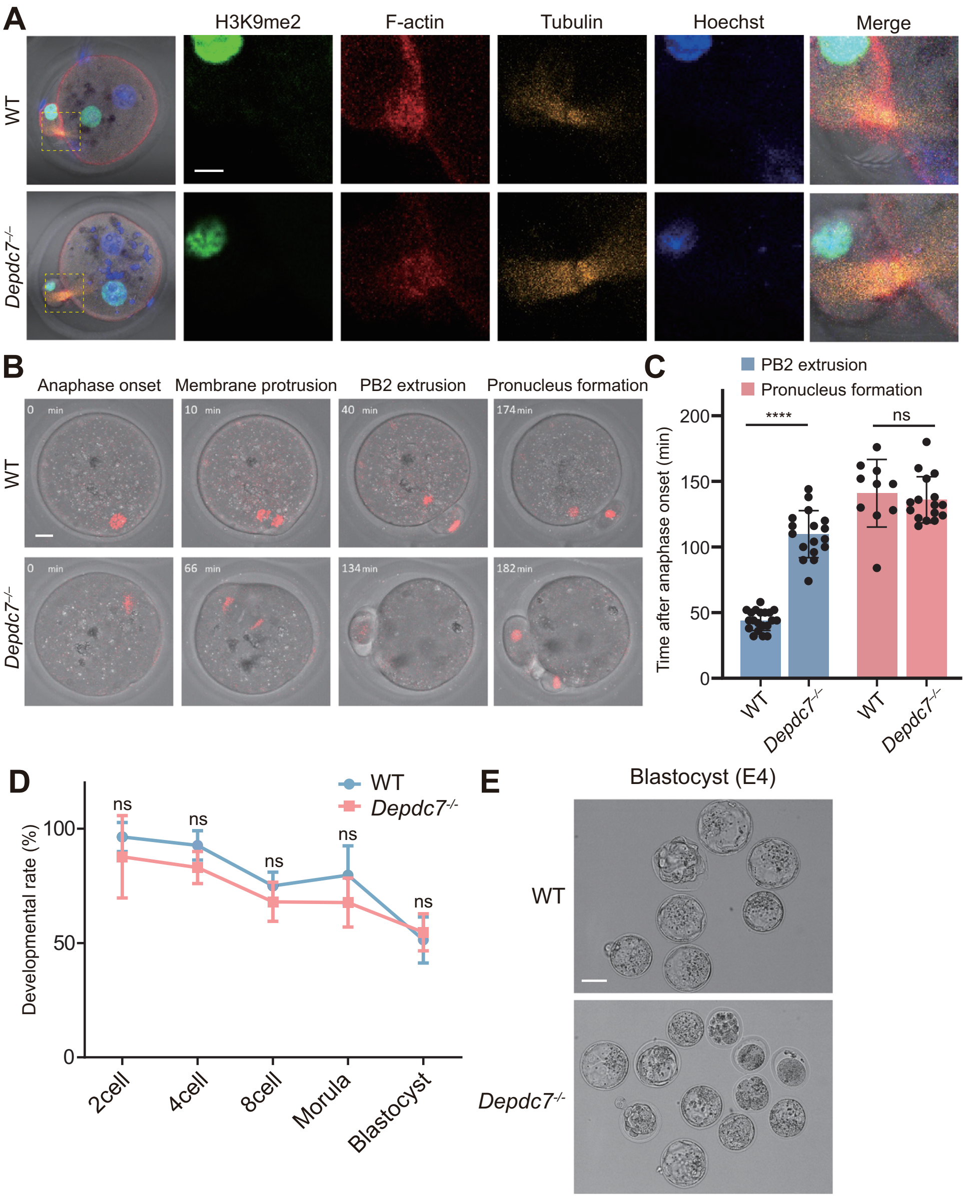

Figure EV4. *Depdc7*$^{-/-}$ oocytes can develop into blastocysts.

(**A**) Immunofluorescence images of 2PN zygotes from WT and *Depdc7*$^{-/-}$ mice after IVF. The polar body neck region is shown at higher magnification corresponding to Fig. 6B. Red, phalloidin-labeled F-actin; blue, Hoechst-labeled DNA; Orange, FITC-tubulin–labeled microtubules; green, H3K9me2. Scale bar, 5 µm. (**B**) Representative time-lapse images showing chromosome dynamics in WT and *Depdc7*$^{-/-}$ oocyte following anaphase onset during parthenogenetic activation. Red, H2B-mScarlet-labeled chromosomes. Scale bar, 10 µm. (**C**) Quantification of the time required for PB2 extrusion and pronucleus (PN) formation in WT and *Depdc7−/−* oocytes. n = 10–20 oocytes from three independent experiments per group. Data were presented as mean ± SD. $^{****}p = 3.94{*}10^{-12}$, $^{ns}p = 0.5980$. Welch's t-test. (**D**) The ratio of developmental stages between WT ($n = 55$) and *Depdc7*$^{-/-}$ ($n = 50$) embryos after IVF. $^{ns1}p = 0.4758$, $^{ns2}p = 0.1529$, $^{ns3}p = 0.3101$, $^{ns4}p = 0.2796$, $^{ns5}p = 0.6779$, Student's t-test. (**E**) Brightfield images of blastocysts obtained from WT and *Depdc7*$^{-/-}$ mated female mice at 90 h after hCG injection. Scale bar, 50 µm. Source data are available online for this figure.
