# Supplementary material for: The mechanisms of DEPDC7-mediated actin cap establishment and meiosis progression in oocytes
Source: EMBO Rep. 2026 Jun 22;27(15):4412–32. doi: 10.1038/s44319-026-00842-0 (PMC13457618; doi:10.1038/s44319-026-00842-0)
Supplement: Supplementary file 2 [file 44319_2026_842_MOESM1_ESM.pdf]

Appendix

The mechanisms of DEPDC7-mediated actin cap establishment and meiosis progression in oocytes

Contents of Appendix:

Appendix Figure S1………………..page 1

Appendix Figure S2………………..page 2

Appendix Figure S3………………..page 3

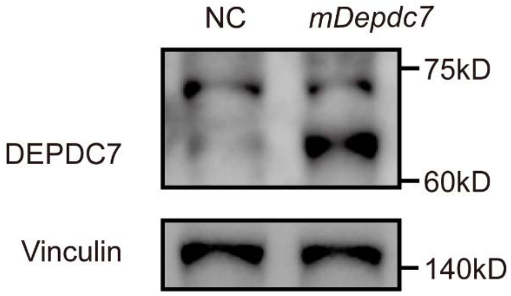

Appendix Figure S1. Overexpression Depdc7 mRNA in GV oocyte. Immunoblot of DEPDC7 and vinculin in WT GV oocytes that overexpressed *Depdc7* mRNA.

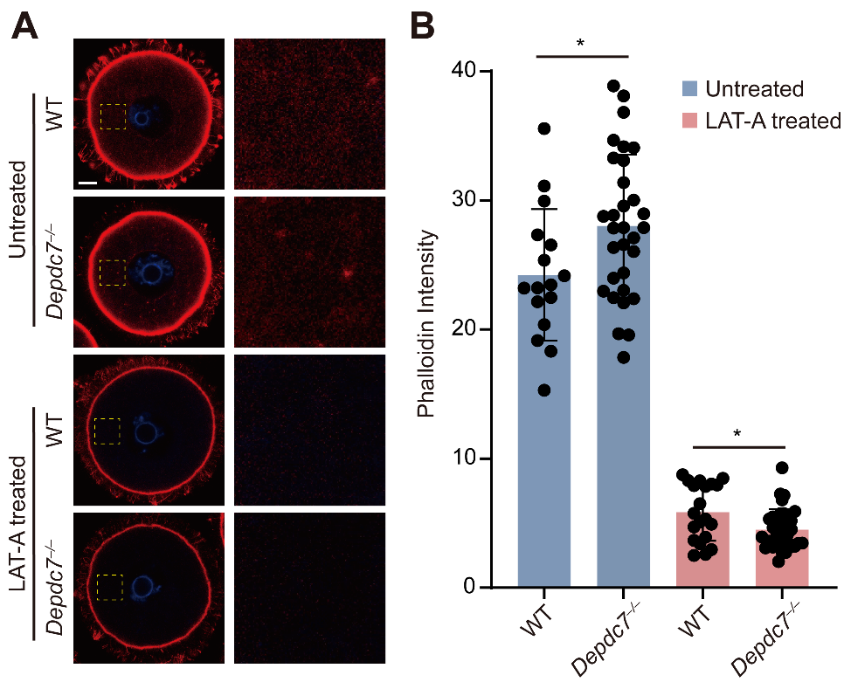

Appendix Figure S2. Cytoplasmic F-actin is not affected in *Depdc7*$^{-/-}$ oocytes. (A) Immunofluorescence staining of GV oocytes treated with or without LAT-A from WT and *Depdc7*$^{-/-}$ mice. The right panel shows a magnified view of the region outlined by the yellow box. Red, phalloidin-labeled F-actin; blue, Hoechst-labeled DNA. Scale bar, 10 μm. (B) Quantification of cytoplasmic F-actin density. The mean intensity of the phalloidin signal was measured in a cytoplasmic 15×15 μm region. n = 16–33 oocytes from three independent experiments per group. $^{*1}P = 0.0348$, $^{*2}P = 0.0165$, Student's t test.

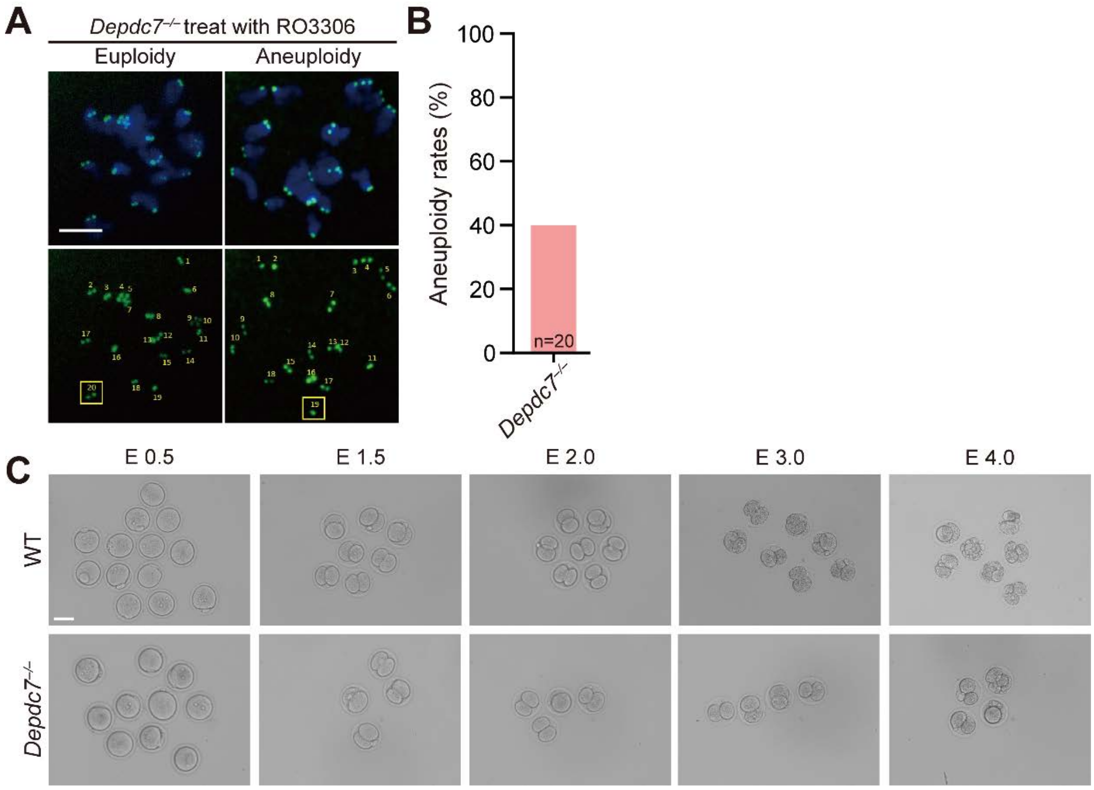

Appendix Figure S3. Oocyte treated with RO3306 affect its cytoplasmic maturation during anaphase I. (A) Chromosome and centromere staining of *Depdc7*$^{-/-}$ (n=20) oocyte treated with RO3306. Scale bar, 5 µm. (B) Quantification of aneuploidy rate in (A). (C) Representative brightfield images of embryos at different time points during cleavage-stage development. The embryos were derived from oocytes that extruded the first polar body following RO3306 treatment and were subsequently fertilized. Scale bar, 50 µm.
