# Supplementary material for: The mechanisms of DEPDC7-mediated actin cap establishment and meiosis progression in oocytes
Source: EMBO Rep. 2026 Jun 22;27(15):4412–32. doi: 10.1038/s44319-026-00842-0 (PMC13457618; doi:10.1038/s44319-026-00842-0)
Supplement: Supplementary file 3 [file 44319_2026_842_MOESM2_ESM.pdf]

The mechanisms of DEPDC7-mediated actin cap establishment and meiosis progression in oocytes

Jian Mu, Siyuan Xie, Lin Zhao, Yang Zeng, Hao Gu, Zhiqi Pan, Ran Yu, Zhihua Zhang, Tianyu Wu, Weijie Wang, Lei Wang, and Qing Sang

Corresponding author(s): Qing Sang (sangqing@fudan.edu.cn) , Lei Wang (wangleiwanglei@fudan.edu.cn)

Review Timeline:

Submission Date:	22nd Oct 25
Editorial Decision:	15th Dec 25
Revision Received:	13th Mar 26
Editorial Decision:	6th May 26
Revision Received:	12th May 26
Accepted:	1st Jun 26

Editor: Kurt Weir

Transaction Report:

(Note: With the exception of the correction of typographical or spelling errors that could be a source of ambiguity, letters and reports are not edited. Depending on transfer agreements, referee reports obtained elsewhere may or may not be included in this compilation. Referee reports are anonymous unless the Referee chooses to sign their reports.)

Dear Prof. Sang

Thank you for the submission of your research manuscript to our journal. We have now received the full set of referee reports that is copied below.

As you have seen, the referees acknowledge that the findings are interesting and that the conclusions are overall supported by the data presented but they also raise a number of concerns and have suggestions how to further strengthen the data, particularly an explanation of how DEPDC7 was selected for investigation and a better description of the rescue phenotype seen with R03306 treatment.

Given these constructive comments, we would like to invite you to revise your manuscript with the understanding that the referee concerns (as detailed above and in their reports) must be fully addressed and their suggestions taken on board. Please address all referee concerns in a complete point-by-point response. Acceptance of the manuscript will depend on a positive outcome of a second round of review. It is EMBO Reports policy to allow a single round of revision only and acceptance or rejection of the manuscript will therefore depend on the completeness of your responses included in the next, final version of the manuscript.

We realize that it is difficult to revise to a specific deadline. In the interest of protecting the conceptual advance provided by the work, we recommend a revision within 3 months (March 15th). Please discuss the revision progress ahead of this time with the editor if you require more time to complete the revisions.

I am also happy to discuss the revision further via e-mail or a video call, if you wish.

You can either publish the study as a short report or as a full article. For short reports, the revised manuscript should not exceed 27,000 characters (including spaces but excluding materials & methods and references) and 5 main plus 5 expanded view figures. The results and discussion sections must further be combined, which will help to shorten the manuscript text by eliminating some redundancy that is inevitable when discussing the same experiments twice. For a normal article there are no length limitations, but it should have more than 5 main figures and the results and discussion sections must be separate. In both cases, the entire materials and methods must be included in the main manuscript file.

================
IMPORTANT NOTE:

We perform an initial quality control of all revised manuscripts before re-review. Your manuscript will FAIL this control and the handling will be delayed IN CASE the following APPLIES:

1) A data availability section providing access to data deposited in public databases is missing. If you have not deposited any data, please add a sentence to the data availability section that explains that.

2) Your manuscript contains statistics and error bars based on n=2. Please use scatter blots in these cases. No statistics should be calculated if n=2.

When submitting your revised manuscript, please carefully review the instructions that follow below. Failure to include requested items will delay the evaluation of your revision.

================

When submitting your revised manuscript, we will require:

1) a .docx formatted version of the manuscript text (including legends for main figures, EV figures and tables). Please make sure that the changes are highlighted to be clearly visible.

2) individual production quality figure files as .eps, .tif, .jpg (one file per figure).
Please download our Figure Preparation Guidelines (figure preparation pdf) from our Author Guidelines pages https://link.springer.com/journal/44319/submission-guidelines for more info on how to prepare your figures.

3) a .docx formatted letter INCLUDING the reviewers' reports and your detailed point-by-point responses to their comments. As part of the EMBO Press transparent editorial process, the point-by-point response is part of the Review Process File (RPF), which will be published alongside your paper.

4) a complete author checklist, which you can download from our author guidelines

(<https://link.springer.com/journal/44319/submission-guidelines>). Please insert information in the checklist that is also reflected in the manuscript. The completed author checklist will also be part of the RPF.

5) Please note that all corresponding authors are required to supply an ORCID ID for their name upon submission of a revised manuscript (<https://orcid.org/>). Please find instructions on how to link your ORCID ID to your account in our manuscript tracking system in our Author guidelines
(<https://link.springer.com/journal/44319/submission-guidelines#authorshipguidelines >)

6) We replaced Supplementary Information with Expanded View (EV) Figures and Tables that are collapsible/expandable online. A maximum of 5 EV Figures can be typeset. EV Figures should be cited as 'Figure EV1, Figure EV2" etc... in the text and their respective legends should be included in the main text after the legends of regular figures.

- For the figures that you do NOT wish to display as Expanded View figures, they should be bundled together with their legends in a single PDF file called *Appendix*, which should start with a short Table of Content. Appendix figures should be referred to in the main text as: "Appendix Figure S1, Appendix Figure S2" etc. See detailed instructions regarding expanded view here:
<https://link.springer.com/journal/44319/submission-guidelines#expandedview>

- Additional Tables/Datasets should be labeled and referred to as Table EV1, Dataset EV1, etc. Legends have to be provided in a separate tab in case of .xls files. Alternatively, the legend can be supplied as a separate text file (README) and zipped together with the Table/Dataset file.

7) Before submitting your revision, primary datasets (and computer code, where appropriate) produced in this study need to be deposited in an appropriate public database (see < https://link.springer.com/journal/44319/submission-guidelines#dataavailability >).

Please remember to provide a reviewer password if the datasets are not yet public.

The accession numbers and database should be listed in a formal "Data Availability " section (placed after Materials & Method) that follows the model below (see also < https://link.springer.com/journal/44319/submission-guidelines#dataavailability >). Please note that the Data Availability Section is restricted to new primary data that are part of this study.

Data availability

The datasets (and computer code) produced in this study are available in the following databases:

- RNA-Seq data: Gene Expression Omnibus GSE46843 (https://www.ncbi.nlm.nih.gov/geo/query/acc.cgi?acc=GSE46843)
- [data type]: [name of the resource] [accession number/identifier/doi] ([URL or identifiers.org/DATABASE:ACCESSION])

*** Note - All links should resolve to a page where the data can be accessed. ***

8) At EMBO Press we ask authors to provide source data for the main manuscript figures. You will receive a separate email with instructions for providing source data with your revised manuscript, including how to upload and organize the files.

Additional information on source data and instruction on how to label the files are available <
https://link.springer.com/journal/44319/submission-guidelines#sourcedata >

9) The journal requires a statement specifying whether or not authors have competing interests (defined as all potential or actual interests that could be perceived to influence the presentation or interpretation of an article). In case of competing interests, this must be specified in your disclosure statement. Further information: https://link.springer.com/partners/embo-press/editorial-policies

10) Figure legends and data quantification:
The following points must be specified in each figure legend:

- the name of the statistical test used to generate error bars and P values,
- the EXACT p-values,
- the number (n) of independent experiments (please specify technical or biological replicates) underlying each data point,
- the nature of the bars and error bars (s.d., s.e.m.)

- If the data are obtained from n {less than or equal to} 5, show the individual data points in addition to the SD or SEM.
- If the data are obtained from n {less than or equal to} 2, use scatter blots showing the individual data points.

Discussion of statistical methodology can be reported in the materials and methods section, but figure legends should contain a

basic description of n, P and the test applied.

See also the guidelines for figure legend preparation: https://link.springer.com/journal/44319/submission-guidelines#figureformat

- Please also include scale bars in all microscopy images and define their size in the legend, not the image.

11) Our journal encourages inclusion of *data citations in the reference list* to directly cite datasets that were re-used and obtained from public databases. Data citations in the article text are distinct from normal bibliographical citations and should directly link to the database records from which the data can be accessed. In the main text, data citations are formatted as follows: "Data ref: Smith et al, 2001" or "Data ref: NCBI Sequence Read Archive PRJNA342805, 2017". In the Reference list, data citations must be labeled with "[DATASET]". A data reference must provide the database name, accession number/identifiers and a resolvable link to the landing page from which the data can be accessed at the end of the reference. Further instructions are available at < https://link.springer.com/journal/44319/submission-guidelines#referencesformat>.

12) All Materials and Methods need to be described in the main text using our 'Structured Methods' format. According to this format, the Methods section includes a Reagents and Tools Table (listing key reagents, experimental models, software and relevant equipment and including their sources and relevant identifiers) followed by a Methods and Protocols section describing the methods, ideally using a step-by-step protocol format. The aim is to facilitate adoption of the methodologies across labs. Please download and fill our Reagents and Tools Table template (.docx), which you can find in our author guidelines: https://link.springer.com/journal/44319/submission-guidelines#structuredmethods.

When submitting your revised manuscript, please do not include the Reagents and Tools Table in the Methods section of the manuscript but upload it as a separate file choosing the file type "Reagent Table".
An example of a Method paper with Structured Methods can be found here:
https://link.springer.com/article/10.15252/msb.20178071

13) As part of the EMBO publication's Transparent Editorial Process, EMBO Reports publishes online a Review Process File to accompany accepted manuscripts. This File will be published in conjunction with your paper and will include the referee reports, your point-by-point response and all pertinent correspondence relating to the manuscript.

You are able to opt out of this by letting the editorial office know (emboreports@embo.org). If you do opt out, the Review Process File link will point to the following statement: "No Review Process File is available with this article, as the authors have chosen not to make the review process public in this case."

We would also welcome the submission of cover suggestions, or motifs to be used by our Graphics Illustrator in designing a cover.

I look forward to seeing a revised form of your manuscript when it is ready and please let me know if you have questions or comments regarding the revision.

Yours sincerely,

Kurt Weir
Editor
EMBO Reports

Referee #1:

The manuscript by Drs. Lei Wang, Qing Sang, and colleagues describes a new role for the poorly understood protein DEPDC7 in the poorly understood process of F-actin cap formation in the cortical region adjacent to the spindle during meiosis I in mammalian oocytes. It has previously been shown that formation of the F-actin cap is initiated by Ran-GTP as the meiotic spindle approaches the oocyte cortex. Ran-GTP initiates a process of F-actin assembly that depends on CDC42, NWASP, and ARP2/3, as well as cortical remodeling through dephosphorylation of Ezrin/Radixin/Moesin (ERM). How the Ran-GTP signal is transduced to the downstream effectors is unknown.

Beginning with the observation that DEPDC7 co-localizes with F-actin in the cortical cap in mouse oocytes, the authors show that Depdc7 is required for female fertility and polar body extrusion during meiosis I. Elegant molecular epistasis experiments demonstrated that Depdc7 is upstream of CDC42, NWASP, ARP2, and ERM phosphorylation and downstream of Ran-GTP. DEPDC7 still associates with the cap region following F-actin depolymerization with Latrunculin A, likely due to membrane anchoring via an N-terminal DEP domain.

The manuscript is clearly written and most arguments are clearly laid out. The data described are of high quality, the experiments are generally well done and include adequate controls, and the claims are novel and convincing. A Pubmed search for Depdc7 results in eight publications, none of which mention actin, WASP, Arp2, etc. Thus, this paper has identified a previously unknown role for DEPDC7 that will likely be of great interest to researchers studying other known roles, for example as a tumor suppressor in hepatoma cells. More importantly, the work begins to shed light on how the Ran-GTP signal is transmitted to the F-actin regulatory machinery at the cortex. More clearly needs to be done to fully understand the process, but the single key message that is the focus of this paper provides an exciting starting point.

Although I believe that this manuscript will be of interest to the readership of EMBO Reports, I do think there is room for improvement. However, most of my suggestions are minor and aimed at improving the clarity of the manuscript rather than requesting additional data.

Major Comments:

1. Lines 81-82, 235-236: In my opinion, the beginning and end of the results section were both somewhat confusing. Although it is not a huge problem to leave it out if there is good reason, it would help ease the reader into the story to provide one or two sentences regarding what motivated the authors to look at DEPDC7 and actin cap localization in oocytes in the first place. As currently written, the first sentence of the Results section made me do a literature search to determine whether the colocalization was first shown here or was previously published.

2. Lines 212-230: I found the logic and rationale laid out in the final paragraph of the results section to be hard to follow. After reading the discussion (Lines 268-286), I felt I understood these experiments somewhat better because certain points are stated more clearly there (for example, "inactivation of CDK1 is sufficient to enable oocyte cytokinesis" is easier to understand than "cytoplasmic CDK1 activity plays a decisive role in cytokinesis, meaning that inhibition of CDK1 activity is sufficient to drive oocyte division." Because the finding is tangential to the main message of the paper, it may be reasonable to remove it from the manuscript, but if it kept in the paper, it needs to be explained more clearly and carefully.

3. Lines 100-101: The "Litters size" / "Fertility assay" shown in Fig. 2B and Fig S1C should be described in the methods.

4. Figure 3A and elsewhere: The methods used to create the intensity traces needs to be clearly described in the methods, and the figure legend should explain how the intensities are normalized (i.e. what does an intensity of 1.0 mean?). This is important because at first glance, the intensity traces in Fig 1A, for example, seem to show that the F-actin cap is equally bright in WT and Depdc7 mutants, but the cytoplasmic F-actin signal is much higher in the mutants than in WT. I assume this is because the brightest region along the linescan was normalized to 1.0 and the darkest region was normalized to 0. Because the F-actin cap signal is dim in the mutant, normalizing this to 1 makes the cytoplasmic signal appear brighter. If this is correct, it should be clearly explained.

5. Lines 156-158, 382-383: If I understand the LatA experiment correctly, the drug was added after the DEPDC7 "patch" would have formed, so this experiment shows that F-actin is not required to _maintain_ DEPDC7 localization but does not address whether F-actin is required for the initial formation of the DEPDC7-enriched region. It would be interesting to know the answer, but at the very least, this should be clearly stated in the results and/or discussion.

6. Figure 3A and elsewhere: the legend clearly states that the red line in the traces under Fig 3A show the F-actin intensity. What the blue line shows is not stated. It should be said somewhere that in all traces, the color of the line matches the color in which the staining is shown.

7. Figure 4B,C: it would be nice to see panels showing only F-actin staining, since it is a bit difficult to see this in the merge. This could be included in a supplemental figure.

8. Figure S3A: I do not understand what "Developmental rate (%)" and "The ratio of developmental stages between WT and Depdc7-/- embryos..." means. Please describe in more detail how Developmental Rate (%) is measured.

9. Line 258: I do not understand the "positive feedback" model, even after referring to figure 8.

10. Lines 268-286: How accurate is the restoration of MI chromosome segregation following CDK1 inactivation by RO3306? Does the first polar body inherit one homolog from each chromosome (i.e. is the oocyte haploid?)? Does RO3306 treatment followed by IVF result in the formation of a diploid embryo that develops further than untreated controls? I'm trying to better understand the functional significance of the observation.

Minor comments:

1. Line 47: If true, introduce FMN2 as a formin.

2. Lines 86-87: I wondered immediately whether it was possible to tell whether DEPDC7 signal increased before, at the same time as, or after the F-actin signal. This question was in my mind the entire time I read the paper until I found the answer in Fig 5E. It might be helpful to move 5E into figure 1, or, alternatively, to foreshadow this finding and say "see below."

3. Line 113: AI is defined as Anaphase I in the text, but this is easy to miss. It would help to also define this in the legend for Fig. 2F.

4. Line 114, 124, etc: I found "re-fusion" to be a confusing description of the events following meiosis I. My initial understanding was that somehow the homologs that separated in anaphase I came back together to form bivalents. Clearly, this does not happen. Instead, the separated homologs move back together and form a cluster. As far as I can tell, nothing ever becomes fused. If this is correct, I think better wording can be found.

5. Figure 3B: the label next to the red box under the figure is missing (i.e. to the left of the blue box labeled "Hoechst").

Referee #2:

To achieve haploidization of the maternal genome, oocytes must execute two highly asymmetric meiotic divisions to discard excess chromosomes in the first (PB1) and second (PB2) polar bodies. Polar body formation requires extensive reorganisation of the oocyte cortex, including the build up of a thick F-actin cap, under the control of the polarized Cdc42/NWASP/Arp2/3 pathway. It has been demonstrated that this cortical polarization is triggerd from a distance by the maternal chromosomes, by means of a gradient of Ran-GTP. However, the mechanism by which Ran activates the cortical polarization pathway remains to be elucidated. Here, Jian Mu and colleagues show that DEPDC7 is a membrane-bound protein that accumulates in the polarized cortical region when the meiotic spindle approaches the cortex. DEPDC7 acts as a key intermediate in driving Ran-induced cortical polarization, thereby promoting first polar body (PB1) extrusion. Deletion of the Depdc7 gene leads to PB1 extrusion failure, the production of triploid embryos, and female infertility.
This interesting work provides valuable new insight into the molecular mechanism of oocyte asymmetric division, with the discovery of DEPDC7 as a novel, key intermediate in Ran-induced cortical polarization. The DEP domain protein DEPDC7 is poorly characterized, and this study linking DEPDC7 to cell polarization, actin remodeling and asymmetric cell division is of general interest for the cell biology community.
The proposed model of DEPDC7 acting as an early effector in the cortical polarization pathway is fairly supported by experimental data, including immuno-localization showing clear cortical polarization of DEPDC7, and genetic invalidation leading to polarity loss and a striking triploidy phenotype. However, the rationale for PB1 and PB2 extrusion being regulated differently remains obscure. In particular, the PB1 extrusion failure phenotype should be more thoroughly characterized ; also, the evidence for PB2 extrusion being unaltered should be described in more details.

Major comments :
1- It is unclear why PB1 extrusion failed in Depdc7-/- oocytes despite normal progression to anaphase-I. Arguably, the absence of membrane protrusion would preclude the formation of a regular polar body. However, MI oocytes are notoriously prone to symmetrical cleavage when the anaphase-I spindle is too far from the cortex to initiate a protrusion (eg., failure of spindle migration). Similarly, Arp2/3-inhibited oocytes were shown to undergo symmetrical cytokinesis - Sun et al 2011 https://doi.org/10.1371/journal.pone.0018392). Here, oocytes lacking an actin cap failed to emit PB1, but showed no sign of symmetrical cleavage, pointing to a possible defect in the cytokinesis machinery. This scenario seems unlikely however because cytokinesis did happen in MII-like oocytes, and also in the CDK1-inhibited oocytes. So the question becomes : why did cytokinesis failed in anaphase-I oocytes lacking an actin cap ? This question should be investigated more thoroughly. Intriguingly, the time-lapse images in Figure 2H and Movie S2 reveal that the spindle in the Depdc7-/- oocyte experienced a significant shift in its location at the time of anaphase-I, suggesting that the anaphase-I spindle may not be properly anchored to the cortex, precluding the establishment of a stable cytokinesis ring. Alternatively, PB1 failure may also arise from a short-lived anaphase-I configuration, leaving too little time to establish the cytokinesis signal (which originates from the central spindle). These informations unfortunately cannot be recovered from the provided images/movies which have very low temporal resolution. Live recording of anaphase-I with better temporal resolution should be attempted, starting at e.g. NEBD+6.5h, with images taken every 1 minute or so. This live imaging of the anaphase-I process would be extremely meaningful as it would provide informations on : 1) whether PB1 protruded or not, 2) the dynamics of chromosomes during anaphase, i.e. the duration of anaphase, and 3) whether the anaphase-I spindle is stably anchored or remains mobile.

2- It is unclear whether PB2 cytokinesis was successful in MII-like oocytes. In Figure 6A (brightfield), cytokinesis seems incomplete in some embryos and it is unclear whether the "second" PB was eventually fully extruded. The authors should consider staining the spindle and the cortical acto-myosin ring to gain further evidence for unalterd cytokinesis. Further, MII-like oocytes could be artificially activated (i.e., $SrCl_2$) in order to monitor the PB2 extrusion process in more details and reveal how these oocytes lacking an actin cap managed to achieve symmetry breaking and PB2 extrusion.

3- Figure 6B is not very meaningful because the pronuclei and the cortical actin layer are not visible. Here, Z-stacks across the entire cell volume, coupled with differential staining of male and female pronuclei, would help in demonstrating that fertilization

produced zygotes containing one single female PN and one male PN.

4- Figure 1 : GV oocytes seem to show faint cortical staining for DEPDC7. However it is stated that DEPDC7 expression starts after NEBD (Figure 8, legend). The possibility that DEPDC7 is already expressed (low level) and localized over the oocyte cortex before maturation starts, should be clarified. An immunoblot experiment using a higher number of GV oocytes may reveal a low level expression of DEPDC7.

5- It is unfortunate that the DEPDC7 immunoblots show two bands behaving differently, one of which being referred to as unspecific. To provide further evidence on band specificity, a similar immunoblot should be realized using oocytes overexpressing DEPDC7 - the unspecific band should not vary, while the specific band should increase.

6- One major conclusion of the study is that establishment of the polarized actin cap is essential for PB1 extrusion. However, it is also shown that PB1 extrusion can be rescued (Figure 7) in the absence of DEPDC7, i.e. presumably in the absence of an actin cap. Also, the authors earlier stated that the actin cap was not the "decisive factor" (line 221). These apparently conflicting statements need clarification. In particular, the rescue experiment (RO3306) should be repeated with detection of F-actin, to find out whether the polarized actin cap is indeed absent, or whether it has been rescued.

Minor comments :
1- line 81 (Results) : The authors should briefly explain why they studied DEPDC7 ;
2- Figure 1A,B : the phalloidin signal should be enhanced a little for better visualization of the cortical F-actin layer - for instance, cortical actin is almost invisible in 2PN, 2-cell and 8-cell embryos ;
3- line 124,126 : the authors probably refer to homologs rather than sister chromatids
4- Line 257 : the authors mention a positive feedback between F-actin and DEPDC7, however experimental evidence for this have not been provided;
5- Line 287 : contradictory statements : Depdc7 is a tumor suppressor ... and promotes liver cancer
6- Methods : please indicate the host species for the primary antibodies ; indicate the fluorophore for phalloidin ; indicate how GV oocytes were maintained in prophase arrest ; the methodology for proteomic data is missing.

Referee #3:

In this study Mu J et al reported a new factor DEPDC7 for the actin assembly in oocytes. The authors showed that DEPDC7 depletion caused the oocyte maturation defects, which is due to the failure of actin-based cytokinesis, while DEPDC7 responded Ran-GTP activity for actin nucleators. Overall, this study filled a gap between Ran GTP and actin nucleators for actin-based oocyte cytokinesis. The review has the following suggestions to improve the study:

1. The authors showed that DEPDC7 regulates actin assembly in oocytes, while actin assembly is conserved in most cell types. In oocytes, the regulation of cytokinesis is similar with other somatic cells, the difference is the actin cap formation, which is due to the meiotic spindle migration. However, DEPDC7 showed no expression in other organs but specifically expressed in oocyte and embryo, but DEPDC7 associated with Ran and Cdc42, the general actin regulators. Why DEPDC7 coordinates with conserved molecules but play unique roles in oocytes should be explained.
2. DEPDC7 "co-localized" with actin, but not "the polarity region", since it not just localized at the actin cap but at the whole oocyte cortex.
3. The authors showed that depletion of DEPDC7 caused the failure of polar body extrusion, but with no big polar bodies, which means that the spindle/chromosome migration is normal, and cytoplasmic actin may be not affected. Cytoplasmic actin examination should be done to confirm this.
4. How Ran-GTP determined DEPDC7 localization, and how DEPDC7 regulated CDC42 to the actin cap are now explained. The potential mechanism should be explored to fill the gap.
5. The errors should be provided for the number of chromosomes Fig 6F.
6. The spindle positioning for the actin cap formation in the diagram of Fig 8 is not correct. The spindle migrates to the cortex, and the pole is faced to the actin cap, but there is no rotation of spindle in late MI stage, since it only occurred after the polar body extrusion.

Referee #1:

The manuscript by Drs. Lei Wang, Qing Sang, and colleagues describes a new role for the poorly understood protein DEPDC7 in the poorly understood process of F-actin cap formation in the cortical region adjacent to the spindle during meiosis I in mammalian oocytes. It has previously been shown that formation of the F-actin cap is initiated by Ran-GTP as the meiotic spindle approaches the oocyte cortex. Ran-GTP initiates a process of F-actin assembly that depends on CDC42, NWASP, and ARP2/3, as well as cortical remodeling through dephosphorylation of Ezrin/Radixin/Moesin (ERM). How the Ran-GTP signal is transduced to the downstream effectors is unknown.

Beginning with the observation that DEPDC7 co-localizes with F-actin in the cortical cap in mouse oocytes, the authors show that Depdc7 is required for female fertility and polar body extrusion during meiosis I. Elegant molecular epistasis experiments demonstrated that Depdc7 is upstream of CDC42, NWASP, ARP2, and ERM phosphorylation and downstream of Ran-GTP. DEPDC7 still associates with the cap region following F-actin depolymerization with Latrunculin A, likely due to membrane anchoring via an N-terminal DEP domain.

The manuscript is clearly written and most arguments are clearly laid out. The data described are of high quality, the experiments are generally well done and include adequate controls, and the claims are novel and convincing. A Pubmed search for Depdc7 results in eight publications, none of which mention actin, WASP, Arp2, etc. Thus, this paper has identified a previously unknown role for DEPDC7 that will likely be of great interest to researchers studying other known roles, for example as a tumor suppressor in hepatoma cells. More importantly, the work begins to shed light on how the Ran-GTP signal is transmitted to the F-actin regulatory machinery at the cortex. More clearly needs to be done to fully understand the process, but the single key message that is the focus of this paper provides an exciting starting point.

Although I believe that this manuscript will be of interest to the readership of EMBO Reports, I do think there is room for improvement. However, most of my suggestions are minor and aimed at improving the clarity of the manuscript rather than requesting additional data.

Response: We thank the referee for the very positive evaluation of our study, which we believe will greatly improve the quality of our manuscript.

Major Comments:

1. Lines 81-82, 235-236: In my opinion, the beginning and end of the results section were both somewhat confusing. Although it is not a huge problem to leave it out if there is good reason, it would help ease the reader into the story to provide one or two sentences regarding what motivated the authors to look at DEPDC7 and actin cap localization in oocytes in the first place. As currently written, the first sentence of the Results section made me do a literature search to determine whether the colocalization was first shown here or was previously published.

Response: We thank the Referee for this important suggestion. Research on Depdc7 began with a systematic investigation into its high expression in oocytes, but for which no relevant studies were available. We hope to discover the physiological functions of these highly expressed genes in oocyte maturation. Thus, we performed immunofluorescence for a group of proteins with relatively high expression in oocytes but their roles in oocyte maturation are rarely explored. Part of these genes were shown in Figure EV1. During the process, we found that DEPDC7 showed specifical localization in the actin cap of MII oocytes. This localization prompted us to speculate that DEPDC7 may play essential roles in the formation of actin cap and oocyte maturation. To be more reasonable, we have added Figure EV1 and text to clarify how Depdc7 initially came to our attention in the revised manuscript (line 80-84):

"Previously, we performed immunofluorescence for a group of proteins with relatively high expression in oocytes but their roles in oocyte development are rarely explored (Fig. EV1). And we found that DEPDC7 showed specifical localization in the actin cap of metaphase I (MI) and metaphase II (MII) oocytes (Fig. 1A). This localization prompted us to speculate that DEPDC7 may play essential roles in the formation of actin cap and oocyte maturation."

2. Lines 212-230: I found the logic and rationale laid out in the final paragraph of the results section to be hard to follow. After reading the discussion (Lines 268-286), I felt I understood these experiments somewhat better because certain points are stated more clearly there (for example, "inactivation of CDK1 is sufficient to enable oocyte cytokinesis" is easier to understand than "cytoplasmic CDK1 activity plays a decisive role in cytokinesis, meaning that inhibition of CDK1 activity is sufficient to drive oocyte division." Because the finding is tangential to the main message of the paper, it may be reasonable to remove it from the manuscript, but if it kept in the paper, it needs to be explained more clearly and carefully.

Response: We thank the Referee for this valuable comment. This part of our study was motivated by an interesting observation: *Depdc7*$^{-/-}$ oocytes fail to extrude PB1 but are able to extrude the PB2. Previous studies have suggested that the actin cap is essential for polar body extrusion, which generally includes both PB1 and PB2 events. In our study, however, we found that PB1 was failed to extrude in the absence of the actin cap, while PB2 extrusion proceeds successfully. This prompted us to explore the underlying mechanism. Literature evidence indicates that CDK1 inactivation alone is sufficient to drive cytoplasmic division in oocytes, which guided our further investigation. These results provide new insights into the molecular mechanisms of polar body extrusion during meiosis: the actin cap appears primarily to facilitate rapid spindle anchoring and membrane protrusion in response to the transient CDK1 inactivation at the end of Meiosis I. Although these findings are relatively preliminary and not directly related to the molecular function of *Depdc7*, we consider them important for understanding the physiological process of polar body extrusion, and therefore have included them as part of the study. In the revised manuscript, we have provided a more detailed discussion to help readers understand the rationale and significance of this part of the study (line 245-256):

"Although *Depdc7*$^{-/-}$ oocytes failed to extrude the PB1, they were still able to extrude the PB2 upon fertilization (Fig. 2C, 6A). This observation suggested that PB1 extrusion depends on the actin cap, whereas PB2 extrusion does not. Previous studies have shown that cytoplasmic CDK1 activity plays a critical role in oocyte cytokinesis: inhibition of CDK1 alone is sufficient to drive oocyte division [18,19]. During normal meiosis, CDK1 activity transiently declines at the MI-to-MII transition and is rapidly reactivated at MII arrest, whereas CDK1 remains inactivated for a prolonged period following fertilization [20–22]. Based on these observations, we hypothesized that the actin cap may not be the decisive factor for PB2 extrusion. Instead, its primary function could be to facilitate timely spindle anchoring and membrane protrusion, particularly during meiosis I when CDK1 inactivation is transient. To explore this, we examined whether CDK1 inhibition during anaphase I could allow *Depdc7*$^{-/-}$ oocytes to complete cytokinesis concurrently with homologous chromosome segregation."

3. Lines 100-101: The "Litters size" / "Fertility assay" shown in Fig. 2B and Fig S1C should be described in the methods.

Response: According to your suggestion. We have added the methods of fertility assay in the revised manuscript (line 389-394):

“For female fertility assay, each 6-8 weeks *Depdc7*$^{-/-}$ female mouse was caged with one 6-8 weeks WT female mouse and one 8-10 weeks male mouse, the number of offspring was recorded. To accurately assess whether *Depdc7*$^{-/-}$ female mouse had mated, vaginal plug checks were performed during the first two months after pairing to ensure that each female had successfully copulated. For male fertility assay, each 8-10 weeks male mouse was caged with two 6-8 weeks WT female mice, and the number of offspring was recorded.”

4. Figure 3A and elsewhere: The methods used to create the intensity traces needs to be clearly described in the methods, and the figure legend should explain how the intensities are normalized (i.e. what does an intensity of 1.0 mean?). This is important because at first glance, the intensity traces in Fig 1A, for example, seem to show that the F-actin cap is equally bright in WT and Depdc7 mutants, but the cytoplasmic F-actin signal is much higher in the mutants than in WT. I assume this is because the brightest region along the linescan was normalized to 1.0 and the darkest region was normalized to 0. Because the F-actin cap signal is dim in the mutant, normalizing this to 1 makes the cytoplasmic signal appear brighter. If this is correct, it should be clearly explained.

Response: Yes, your understand is right. To allow comparison between groups, fluorescence intensities were normalized. For most figure and channel, fluorescence intensity along the line-scan was quantified using ZEN software (Zeiss) and exported to Microsoft Excel. For each line-scan, the maximum intensity was normalized to 1.0 and the minimum to 0. Specifically, for normalization in Figure 1C, the mean fluorescence intensity along the entire line scan was set to 1, and the fluorescence intensity at each point was normalized to this value. This information has been added to the Materials and Methods section (line 442-449) and the corresponding figure legends.

“Fluorescent intensity measurement

The direction of the line-scan intensity measurements is shown in the figures. The fluorescence intensity was quantified using ZEN software (Zeiss) and exported into Microsoft Excel for further analysis. For normalization, the minimum intensity value along the line-scan was set to 0 and the maximum value was set to 1.0, and all intermediate values were scaled accordingly. Specifically, for normalization in Figure 1C, the mean fluorescence intensity along the entire line scan was set to 1, and the fluorescence intensity at each point was normalized to this value.”

5. Lines 156-158, 382-383: If I understand the LatA experiment correctly, the drug was

added after the DEPDC7 "patch" would have formed, so this experiment shows that F-actin is not required to _maintain_ DEPDC7 localization but does not address whether F-actin is required for the initial formation of the DEPDC7-enriched region. It would be interesting to know the answer, but at the very least, this should be clearly stated in the results and/or discussion.

Response: Yes, you are correct. The LatA treatment only demonstrates that the maintenance of DEPDC7 polarity does not depend on cortical F-actin. It does not address whether F-actin is required for the initial formation of the DEPDC7-enriched region. To answer this question, cortical F-actin need to be inhibited before spindle migration to the oocyte cortex. Previous studies have shown that the formation of the polarity region requires asymmetrically positioned chromosomes to generate asymmetrical Ran-GTP gradients. However, spindle migration, which is driven by cytoplasmic F-actin dynamics, is necessary for chromosome asymmetry. We do not find a proper way to inhibit cortical F-actin but do not influence the cytoplasmic F-actin dynamics. Thus, inhibiting F-actin would prevent spindle migration, making it technically challenging to answer whether F-actin is required for the initial formation of the DEPDC7-enriched region. We have added in Discussion (line 340-349):

“The study has some limitations. First, our Lat-A experiments could only demonstrate that cortical F-actin is not required for the maintenance of DEPDC7 polarity localization. But we could not directly determine whether F-actin is required for the initial formation of DEPDC7 polarity. Because the formation of the polarity domain depends on asymmetrically positioned chromosomes, which generate an asymmetric Ran-GTP gradient. However, chromosome asymmetry requires spindle migration to the cortex, a process driven by F-actin dynamics. Therefore, inhibiting F-actin disrupts spindle migration and prevents polarity establishment, making it difficult to distinguish a direct role of cortical F-actin in initial formation of DEPDC7 enrichment. Whether F-actin is required for the initial establishment of the DEPDC7-enriched region remains to be determined.”

6. Figure 3A and elsewhere: the legend clearly states that the red line in the traces under Fig 3A show the F-actin intensity. What the blue line shows is not stated. It should be said somewhere that in all traces, the color of the line matches the color in which the staining is shown.

Response: We have checked and updated the figure legends to ensure that all corresponding figures indicate that the blue line represents chromosomes.

7. Figure 4B,C: it would be nice to see panels showing only F-actin staining, since it is a bit difficult to see this in the merge. This could be included in a supplemental figure.

Response: Following your suggestion, all panels corresponding to Figure 4B-C have been compiled and presented as the new Figure EV3A-B.

8. Figure S3A: I do not understand what "Developmental rate (%)" and "The ratio of developmental stages between WT and Depdc7-/- embryos..." means. Please describe in more detail how Developmental Rate (%) is measured.

Response: In our study, the developmental rate refers to the proportion of embryos that reach a given stage relative to the number of normally fertilized eggs (2PN). For example, the cleavage rate is calculated as the number of embryos reaching the 2-cell or 4-cell stage divided by the total number of 2PN embryos, and the blastocyst formation rate is calculated as the number of blastocysts divided by the total number of 2PN embryos. We have added the description in Materials and Methods section (line 413-416):

"Developmental rates of embryos were calculated based on the number of normally fertilized eggs (2PN). It was defined as the proportion of 2PN embryos that reached the given cleavage stage. Data are presented as mean ± SD."

9. Line 258: I do not understand the "positive feedback" model, even after referring to figure 8.

Response: We are sorry for the confusion caused by our description of the "positive feedback" model. The description of the feedback mechanism is an interpretation based on our current findings. However, upon careful reconsideration, we realize that the current experimental evidence is not sufficient to rigorously support the use of this terminology. Therefore, in the revised manuscript, we have modified the description to avoid implying a definitive positive feedback mechanism. The revised text can be found in the Discussion section (lines 288-291):

"We propose that DEPDC7 responds to Ran-GTP signaling and becomes enriched at the plasma membrane adjacent to the chromosomes, where it may facilitate the recruitment of actin cap–associated proteins to the polarized cortex. The coordinated enrichment and assembly of these proteins ultimately give rise to actin cap formation (Fig. 8)."

10. Lines 268-286: How accurate is the restoration of MI chromosome segregation following CDK1 inactivation by RO3306? Does the first polar body inherit one homolog from each chromosome (i.e. is the oocyte haploid?)? Does RO3306 treatment followed

by IVF result in the formation of a diploid embryo that develops further than untreated controls? I'm trying to better understand the functional significance of the observation.

Response: Following your suggestion, we have re-evaluated chromosome segregation following RO3306 treatment. Depdc7$^{-/-}$ oocytes were treated 6 hours after NEBD, and oocytes that had extruded the first polar body were collected. After monastrol treatment, oocytes were fixed and stained for centromere using anti-CREST antibody. Centromeres in the cytoplasm were counted to determine oocyte ploidy. Our analysis revealed an aneuploidy rate of approximately 40% (Appendix Figure S3A-B), which is substantially higher than the ~3% reported for naturally occurring MII mouse oocytes (Dong J. et al. Cell Discovery, 2023). This elevated aneuploid rate may be explained as following: although oocytes had entered late MI 6 hours after NEBD, some incorrect kinetochore–microtubule attachments were still present. Premature progression into anaphase I induced by RO3306 treatment may therefore improve chromosome segregation errors, resulting in the observed high aneuploidy. Nevertheless, approximately 60% of the oocytes remained diploid, indicating that the first polar body inherited one set of homologous chromosomes. It is important to note that MII oocytes are diploid (2n) rather than haploid, consistent with normal meiotic progression.

The method for RO3306 treatment of MI oocytes was adapted from a previous study (Wei Z. et al. Nat Commun, 2018). However, to our knowledge, no studies have reported performing IVF after RO3306 treatment. Therefore, we first attempted this approach using WT oocytes. Unfortunately, MII oocytes obtained via this method exhibited poor fertilization and embryonic development, and all embryos failed to reach the 4-cell stage. We also performed a similar attempt using Depdc7$^{-/-}$ oocytes, but again 4-cell could not be obtained (Appendix Figure S3C). This likely reflects compromised cytoplasmic maturation caused by premature progression into MII induced by RO3306, which reduces the oocytes' fertilization potential and developmental competence.

Furthermore, the purpose of RO3306 treatment was to address why Depdc7$^{-/-}$ oocytes fail to extrude the PB1 while still being able to extrude the PB2 after fertilization. Our intention was not to use RO3306 as a rescue strategy, but rather as a mechanistic tool to test whether CDK1 inhibition could permit oocyte extruding PB1 without actin cap. After confirming that RO3306 treatment allowed PB1 extrusion in Depdc7$^{-/-}$ oocytes, we did not further investigate potential long-term effects of the treatment. Nevertheless, we agree that the referee's concern is valid. Additional analyses indicate that RO3306 treatment indeed

compromises oocyte quality, including increased aneuploidy and reduced developmental competence.

We have addressed this point in the revised Discussion (line 329-339):

"Furthermore, inhibition of CDK1 by RO3306 allowed Depdc7-/- oocytes to extrude the PB1, this rescue was incomplete. The aneuploidy rate increased to approximately 40%, the fertilization rate was markedly reduced, and almost all embryos arrested before the 4-cell stage (Appendix Fig. S3). The onset of anaphase during meiosis normally requires satisfaction of the spindle assembly checkpoint (SAC), ensuring that all kinetochores are properly attached to spindle microtubules. However, treatment with RO3306 prematurely triggered anaphase onset, thereby bypassing proper checkpoint control. Consequently, some erroneous kinetochore–microtubule attachments may persist and lead to an increased incidence of aneuploidy. Thus, premature progression into anaphase I induced by RO3306 treatment may reflect compromised cytoplasmic maturation of the oocyte, thereby increasing chromosome segregation errors and reducing the fertilization potential and developmental competence of the oocytes."

Minor comments:

1. Line 47: If true, introduce FMN2 as a formin.

Response: We have replaced FMN2 as formin-2 which is considered the critical actin-nucleating protein in mammalian oocytes.

2. Lines 86-87: I wondered immediately whether it was possible to tell whether DEPDC7 signal increased before, at the same time as, or after the F-actin signal. This question was in my mind the entire time I read the paper until I found the answer in Fig 5E. It might be helpful to move 5E into figure 1, or, alternatively, to foreshadow this finding and say "see below."

Response: Thanks for your helpful suggestion. We have moved Fig. 5E to an earlier position in the revised manuscript (now Fig. 1C, line 91-94) so that the temporal relationship between DEPDC7 and F-actin signals is immediately apparent.

3. Line 113: AI is defined as Anaphase I in the text, but this is easy to miss. It would help to also define this in the legend for Fig. 2F.

Response: We found that Figure 2F does not involve anaphase I (AI). We found

that Figure S1F (now Fig. EV2F), Figure 7C, and Figure 8 were involved in definition of AI. We have now added the definition of AI (anaphase I) at these locations to ensure clarity throughout the manuscript.

4. Line 114, 124, etc: I found "re-fusion" to be a confusing description of the events following meiosis I. My initial understanding was that somehow the homologs that separated in anaphase I came back together to form bivalents. Clearly, this does not happen. Instead, the separated homologs move back together and form a cluster. As far as I can tell, nothing ever becomes fused. If this is correct, I think better wording can be found.

Response: We thank the referee for this important suggestion. We agree that the term "re-fusion" may imply re-pairing of homologous chromosomes. To avoid misunderstanding, we have replaced it with "reassembly". We have revised in the manuscript (line 121 and line 140).

5. Figure 3B: the label next to the red box under the figure is missing (i.e. to the left of the blue box labeled "Hoechst").

Response: The missing label next to the red box is "F-actin". We have added in the revised version of Figure 3B. We apologize for the oversight and have carefully rechecked all figures to ensure completeness and accuracy of labeling.

Referee #2:

To achieve haploidization of the maternal genome, oocytes must execute two highly asymmetric meiotic divisions to discard excess chromosomes in the first (PB1) and second (PB2) polar bodies. Polar body formation requires extensive reorganisation of the oocyte cortex, including the build up of a thick F-actin cap, under the control of the polarized Cdc42/NWASP/Arp2/3 pathway. It has been demonstrated that this cortical polarization is triggerd from a distance by the maternal chromosomes, by means of a gradient of Ran-GTP. However, the mechanism by which Ran activates the cortical polarization pathway remains to be elucidated. Here, Jian Mu and colleagues show that DEPDC7 is a membrane-bound protein that accumulates in the polarized cortical region when the meiotic spindle approaches the cortex. DEPDC7 acts as a key intermediate in driving Ran-induced cortical polarization, thereby promoting first polar body (PB1) extrusion. Deletion of the Depdc7 gene leads to PB1 extrusion failure, the production of triploid embryos, and female infertility.

This interesting work provides valuable new insight into the molecular mechanism of oocyte asymmetric division, with the discovery of DEPDC7 as a novel, key intermediate in Ran-induced cortical polarization. The DEP domain protein DEPDC7 is poorly characterized, and this study linking DEPDC7 to cell polarization, actin remodeling and asymmetric cell division is of general interest for the cell biology community.

The proposed model of DEPDC7 acting as an early effector in the cortical polarization pathway is fairly supported by experimental data, including immuno-localization showing clear cortical polarization of DEPDC7, and genetic invalidation leading to polarity loss and a striking triploidy phenotype. However, the rationale for PB1 and PB2 extrusion being regulated differently remains obscure. In particular, the PB1 extrusion failure phenotype should be more thoroughly characterized ; also, the evidence for PB2 extrusion being unaltered should be described in more details.

Response: We thank the referee for the careful evaluation of our manuscript and for the helpful comments and suggestions.

Major comments :

1- It is unclear why PB1 extrusion failed in Depdc7-/- oocytes despite normal progression to anaphase-I. Arguably, the absence of membrane protrusion would preclude the formation of a regular polar body. However, MI oocytes are notoriously prone to symmetrical cleavage when the anaphase-I spindle is too far from the cortex to initiate a protrusion (eg., failure of spindle migration). Similarly, Arp2/3-inhibited

oocytes were shown to undergo symmetrical cytokinesis - Sun et al 2011 https://doi.org/10.1371/journal.pone.0018392). Here, oocytes lacking an actin cap failed to emit PB1, but showed no sign of symmetrical cleavage, pointing to a possible defect in the cytokinesis machinery. This scenario seems unlikely however because cytokinesis did happen in MII-like oocytes, and also in the CDK1-inhibited oocytes. So the question becomes : why did cytokinesis failed in anaphase-I oocytes lacking an actin cap ? This question should be investigated more thoroughly. Intriguingly, the time-lapse images in Figure 2H and Movie S2 reveal that the spindle in the Depdc7-/- oocyte experienced a significant shift in its location at the time of anaphase-I, suggesting that the anaphase-I spindle may not be properly anchored to the cortex, precluding the establishment of a stable cytokinesis ring. Alternatively, PB1 failure may also arise from a short-lived anaphase-I configuration, leaving too little time to establish the cytokinesis signal (which originates from the central spindle). These informations unfortunately cannot be recovered from the provided images/movies which have very low temporal resolution. Live recording of anaphase-I with better temporal resolution should be attempted, starting at e.g. NEBD+6.5h, with images taken every 1 minute or so. This live imaging of the anaphase-I process would be extremely meaningful as it would provide informations on : 1) whether PB1 protruded or not, 2) the dynamics of chromosomes during anaphase, i.e. the duration of anaphase, and 3) whether the anaphase-I spindle is stably anchored or remains mobile.

Response: We thank the referee for the valuable suggestion. Following this advice, we performed higher temporal resolution live imaging of oocytes at 6.5 h after NEBD, acquiring images every 2 minutes. As shown in the time-lapse recordings (movie EV3, EV4), *Depdc7*$^{-/-}$ oocytes were able to enter and maintain anaphase I; however, no membrane protrusion was observed. Meanwhile, the spindle continued to move dynamically beneath the cortex, indicating that the spindle was not stably anchored at the subcortical region. Furthermore, we speculate that spindle anchoring defects alone are unlikely to be the primary cause of PB1 extrusion failure. During PB2 extrusion, we also observed persistent spindle movement after anaphase onset in *Depdc7*$^{-/-}$ oocytes. Nevertheless, these oocytes ultimately formed membrane protrusions and successfully extruded PB2 (Figure EV4B-C, movie EV5, EV6). These observations support our hypothesis that during meiotic anaphase, the actin cap facilitates rapid spindle anchoring and membrane protrusion to ensure timely polar body extrusion. When the actin cap is compromised, this rapid mechanism is disrupted. However, cytokinesis can still proceed, when CDK1 activity declines below a critical threshold (Pomerantz, Y., et al. The FASEB Journal. 2012; Wolf F, et al. Cell Cycle. 2007; Willet AH, et al. Mol Biol Cell. 2018).

We have added the result in revised manuscript (line 125-134)

“Furthermore, we performed higher temporal resolution live imaging of oocytes at 6.5 h after NEBD, acquiring images every 2 minutes to monitor the AI progression in oocytes. In WT oocytes, following anaphase onset, homologous chromosomes segregate rapidly, accompanied by membrane protrusion, resulting in the extrusion of the PB1. In Depdc7$^{-/-}$ oocytes, although homologous chromosomes also segregate promptly upon anaphase onset, the spindle moves within the cytoplasm, indicating that it cannot stably anchor at the cortex in the absence of an actin cap. Notably, one set of chromosomes remained adjacent to the plasma membrane without triggering membrane protrusion, and after a period, the segregated homologous chromosomes reassembled into a new spindle. (movie EV3 and EV4).”

2- It is unclear whether PB2 cytokinesis was successful in MII-like oocytes. In Figure 6A (brightfield), cytokinesis seems incomplete in some embryos and it is unclear whether the "second" PB was eventually fully extruded. The authors should consider staining the spindle and the cortical acto-myosin ring to gain further evidence for unalterd cytokinesis. Further, MII-like oocytes could be artificially activated (i.e., SrCl2) in order to monitor the PB2 extrusion process in more details and reveal how these oocytes lacking an actin cap managed to achieve symmetry breaking and PB2 extrusion.

Response: Thanks for your professional comments. Following your suggestion, we performed immunofluorescence staining on 2PN zygote. As shown in Figure EV4A, a residual F-actin signal can be observed at the polar body neck, while the microtubules have already been severed, indicating that the polar body has been fully separated. It should be emphasized that, in general, if cytokinesis is incomplete, the cells that fail to divide typically regress into a single cell rather than forming two separate cells. As shown in the upper right panel of Figure 6C, the polar body can still be observed at the 2-cell stage in Depdc7$^{-/-}$ embryos, indicating that it can exist as a stable, independent cell. So, the PB2 would be eventually fully extruded.

We also performed live-cell imaging to monitor PB2 extrusion in oocytes artificially activated with $SrCl_2$. As shown in Figure EV4B-C and movie EV5, EV6, in WT oocytes, membrane protrusion occurred shortly after anaphase onset, and the second polar body was extruded at approximately 40 min. In contrast, Depdc7$^{-/-}$ oocytes did not exhibit immediate membrane protrusion following anaphase onset; instead, they experienced a prolonged waiting period, with second polar body extrusion occurring around 110 min, indicating a significant

delay. Despite this difference, the timing of pronucleus formation was comparable between the two groups, occurring at approximately 140 min after anaphase onset. Given that CDK1 activity gradually declines from anaphase to interphase, these observations support our hypothesis that, in the presence of an actin cap, oocytes can extrude the polar body rapidly after anaphase onset, whereas in the absence of an actin cap, polar body extrusion is inhibited until CDK1 activity decreases to a permissive threshold.

We have added in Result section (line 213-226):

"To determine whether fertilization and second polar body extrusion occur normally in *Depdc7*$^{-/-}$ oocytes, we collected 2PN zygotes following IVF. H3K9me2 staining confirmed that *Depdc7*$^{-/-}$ oocytes were normally fertilized and formed one male and one female pronucleus (Fig. 6B). As shown in Fig. EV4A, a residual F-actin signal was detected at the polar body neck, whereas microtubules were no longer present at the intercellular bridge, indicating that the polar body had been fully separated. These results suggest that *Depdc7*$^{-/-}$ oocytes can extrude polar body after fertilization. We next performed live-cell imaging following parthenogenetic activation to monitor the process of polar body extrusion. Membrane protrusion occurred shortly after anaphase onset in WT oocytes, and the second polar body was extruded at approximately 40 min. In contrast, *Depdc7*$^{-/-}$ oocytes failed to exhibit immediate membrane protrusion following anaphase onset, and second polar body extrusion occurred at approximately 110 min. Despite this difference, pronucleus formation occurred at a comparable time point in both groups, at approximately 140 min after anaphase onset (Fig. EV4B-C, movie EV5 and EV6)."

3- Figure 6B is not very meaningful because the pronuclei and the cortical actin layer are not visible. Here, Z-stacks across the entire cell volume, coupled with differential staining of male and female pronuclei, would help in demonstrating that fertilization produced zygotes containing one single female PN and one male PN.

Response: We have revised Figure 6B as following：First, we added tubulin staining and enhanced the actin signal to better visualize the cytoskeletal organization. Second, we replaced the immunofluorescence image of DEPDC7 with H3K9me2 staining, which specifically marks the female pronucleus without labeling the male pronucleus (Ma XS, et al. Sci Rep, 2016), while also enhancing the nuclear signal. In *Depdc7*$^{-/-}$ zygotes, the PB2 exhibits a weak actin signal, and the cytoplasm contains both one male and one female pronucleus.

4- Figure 1 : GV oocytes seem to show faint cortical staining for DEPDC7. However it

is stated that DEPDC7 expression starts after NEBD (Figure 8, legend). The possibility that DEPDC7 is already expressed (low level) and localized over the oocyte cortex before maturation starts, should be clarified. An immunoblot experiment using a higher number of GV oocytes may reveal a low level expression of DEPDC7.

Response: We have corrected Figure 8 and legend. DEPDC7 indeed have expression at GV stage, as shown in Figure 1B. And we have replaced the Figure 1C (now Fig. 1D). In the new figure 1D, a faint band of DEPDC7 can be observed at the GV stage.

5- It is unfortunate that the DEPDC7 immunoblots show two bands behaving differently, one of which being referred to as unspecific. To provide further evidence on band specificity, a similar immunoblot should be realized using oocytes overexpressing DEPDC7 - the unspecific band should not vary, while the specific band should increase.

Response: We thank the referee for this comment. Following your comments, we overexpressed Depdc7 mRNA in oocyte. As shown in Appendix Figure S1, the specific band was increased but the unspecific band was not.

We have added in Result section (line 95-98):

"To confirm the specificity of the DEPDC7 band, mDepdc7 was overexpressed in oocytes. Upon overexpression, the expected DEPDC7 band was specifically restored, whereas the additional band remained unchanged, indicating that it represents a non-specific signal (Appendix Fig. S1)."

6- One major conclusion of the study is that establishment of the polarized actin cap is essential for PB1 extrusion. However, it is also shown that PB1 extrusion can be rescued (Figure 7) in the absence of DEPDC7, i.e. presumably in the absence of an actin cap. Also, the authors earlier stated that the actin cap was not the "decisive factor" (line 221). These apparently conflicting statements need clarification. In particular, the rescue experiment (RO3306) should be repeated with detection of F-actin, to find out whether the polarized actin cap is indeed absent, or whether it has been rescued.

Response: Thanks for your comments. We evaluated cortical actin fluorescence intensity in oocytes treated with RO3306. As shown in Figure 7D, its intensity was markedly lower than that of a typical actin cap of WT oocyte. Actually, there is no conflict. In our study, we found that PB1 extrusion is a balance and synergy between CDK1 activity and actin cap formation. During PB1 extrusion, CDK1 only undergoes a short window of rapid inactivation, during this short period of CDK1 inactivation, actin cap is essential to facilitate the extrusion of PB1; thus, both CDK1 inactivation and actin cap are required for PB1 extrusion in

physiological condition. When we treated *Depdc7*$^{-/-}$ oocytes with RO3306, the CDK1 activity was inhibited for a long time artificially, in this condition, the role of actin cap is less important. However, during PB2 extrusion, CDK1 undergoes sustained and complete inactivation for a long time in a physiological state, this allows cytokinesis to occur even without an actin cap, suggesting actin cap is not a decisive factor for PB2 extrusion. So, our statement of "the actin cap was not the "decisive factor" (line 221)" is referred to PB2 extrusion, not PB1 extrusion. Taken together, there is no conflict for the role of actin cap in PB extrusion.

The result of evaluating cortical actin fluorescence intensity in *Depdc7*$^{-/-}$ oocytes treated with RO3306 was added in revised the manuscript (line 260-261)

"We evaluated cortical actin fluorescence intensity in Depdc7$^{-/-}$ oocytes treated with RO3306. its intensity was markedly lower than that of a typical actin cap of WT oocyte (Fig. 7D)."

To avoid the confusing, we revised the statement (line 251-252):

"Based on these observations, we hypothesized that the actin cap may not be the decisive factor for PB2 extrusion."

Minor comments :
1- line 81 (Results) : The authors should briefly explain why they studied DEPDC7 ;

Response: We thank the referee for this comment. This concern was also raised by Referee #1 major point 1. Briefly, our study was initiated with a systematic investigation of highly expressed proteins in oocytes for which limited functional studies are currently available. The corresponding clarification has been added to the revised manuscript as following (Line 80-84):

"Previously, we performed immunofluorescence for a group of proteins with relatively high expression in oocytes but their roles in oocyte development are rarely explored (Fig. EV1). And we found that DEPDC7 showed specifical localization in the actin cap of metaphase I (MI) and metaphase II (MII) oocytes (Fig. 1A). This localization prompted us to speculate that DEPDC7 may play essential roles in the formation of actin cap and oocyte maturation."

2- Figure 1A,B : the phalloidin signal should be enhanced a little for better visualization of the cortical F-actin layer - for instance, cortical actin is almost invisible in 2PN, 2-cell and 8-cell embryos ;

Response: The brightness and contrast of the phalloidin signal in Figure 1A and

1B have been slightly enhanced to improve the visibility of the cortical F-actin layer. Importantly, this adjustment does not affect the original data. The updated images are now clearer showing cortical F-actin.

3- line 124,126 : the authors probably refer to homologs rather than sister chromatids

Response: We have replaced the term "sister chromatids" with "homologous chromosomes" (line 140 and 142).

4- Line 257 : the authors mention a positive feedback between F-actin and DEPDC7, however experimental evidence for this have not been provided;

Response: Thanks for pointing out this, which will improve the accuracy of our description. As you mentioned, we realize that the current experimental evidence is not sufficient to rigorously support the use of this terminology. Therefore, in the revised manuscript, we have modified the description to avoid implying a definitive positive feedback mechanism. We have instead provided a more cautious and evidence-based interpretation of our findings to better reflect the data presented. The revised text can be found in the Discussion (lines 288-291):

"We propose that DEPDC7 responds to Ran-GTP signaling and becomes enriched at the plasma membrane adjacent to the chromosomes, where it may facilitate the recruitment of actin cap–associated proteins to the polarized cortex. The coordinated enrichment and assembly of these proteins ultimately give rise to actin cap formation (Fig. 8)."

5- Line 287 : contradictory statements : Depdc7 is a tumor suppressor ... and promotes liver cancer

Response: We are sorry for our confusing statements. This statement is meant to express that DEPDC7 functions as a tumor suppressor, and downregulation of DEPDC7 in liver cancer cells contributes to cancer progression. We have revised the statement in the manuscript to clarify this point (line 361-363):

"Two studies reported that DEPDC7 acts as a tumor suppressor, and reduced expression of DEPDC7 in liver cancer cells promotes cancer progression."

6- Methods : please indicate the host species for the primary antibodies ; indicate the fluorophore for phalloidin ; indicate how GV oocytes were maintained in prophase arrest ; the methodology for proteomic data is missing.

Response: We have added the required information in revised manuscript (Reagents and Tools Table and line 398 and line 470-481).

Referee #3:

In this study Mu J et al reported a new factor DEPDC7 for the actin assembly in oocytes. The authors showed that DEPDC7 depletion caused the oocyte maturation defects, which is due to the failure of actin-based cytokinesis, while DEPDC7 responded Ran-GTP activity for actin nucleators. Overall, this study filled a gap between Ran GTP and actin nucleators for actin-based oocyte cytokinesis. The review has the following suggestions to improve the study:

Response: Thanks for your positive feedback and thoughtful comments.

1. The authors showed that DEPDC7 regulates actin assembly in oocytes, while actin assembly is conserved in most cell types. In oocytes, the regulation of cytokinesis is similar with other somatic cells, the difference is the actin cap formation, which is due to the meiotic spindle migration. However, DEPDC7 showed no expression in other organs but specifically expressed in oocyte and embryo, but DEPDC7 associated with Ran and Cdc42, the general actin regulators. Why DEPDC7 coordinates with conserved molecules but play unique roles in oocytes should be explained.

Response: Thanks for your comments. We agree with the referee that Ran and Cdc42 are general actin regulators which is the a conserved signaling pathway. In somatic cells undergoing mitosis, cytokinesis is typically symmetric and the mitotic spindle remains centrally positioned, and therefore cortical actin remodeling occurs relatively uniformly around the cell cortex. In contrast, oocyte meiosis is a highly asymmetric division. During meiosis, the spindle needs to migrate from the center of the oocyte toward the cortex, and anchor to the cortex enable to extrude the polar body. To achieve this asymmetric division, oocytes establish a specialized actin cap signaling pathway that facilitates cortical anchoring and precise positioning of the spindle. This division mode differs fundamentally from symmetric mitotic divisions in somatic cells, thus, oocyte needs specific signaling factors to coordinate conserved actin regulators with the unique structure. In other tissues, DEPDC7 is expressed at relatively low levels compared with oocytes, and whether it performs similar functions in these tissues remains to be investigated.

We have added in the Discussion (line 349-353):

"Second, although DEPDC7 is highly expressed in oocytes, its expression in other tissues appears to be relatively low. To date, functional studies of DEPDC7

remain very limited. Here, we report for the first time that DEPDC7 participates in regulating actin dynamics in oocytes. Whether DEPDC7 has similar functions in other cell types requires further investigation."

2. DEPDC7 "co-localized" with actin, but not "the polarity region", since it not just localized at the actin cap but at the whole oocyte cortex.

Response: We agree that DEPDC7 is not restricted to the polarity region but co-localized with cortical F-actin. Accordingly, we have revised the legend of Figure 1 as following: "DEPDC7 co-localizes with cortical F-actin during oocyte maturation." (line 621)

3. The authors showed that depletion of DEPDC7 caused the failure of polar body extrusion, but with no big polar bodies, which means that the spindle/chromosome migration is normal, and cytoplasmic actin may be not affected. Cytoplasmic actin examination should be done to confirm this.

Response: Thanks for your the helpful suggestion. We performed Phalloidin staining in both WT and Depdc7$^{-/-}$ oocytes, and quantified the cytoplasmic fluorescence intensity. Cytoplasmic actin fluorescence intensity in Depdc7$^{-/-}$ oocytes was not decreased, rather, it was slightly but significantly increased compared to WT controls (Appendix Figure S2). Furthermore, in all Depdc7$^{-/-}$ oocytes shown in Figures, the chromosomes were positioned beneath the cortex. Moreover, our supplementary videos (movie EV2, EV4) demonstrate that chromosomes in Depdc7$^{-/-}$oocytes are able to migrate from the center to the cortex. Together, these observations indicate that cytoplasmic F-actin remains largely unaffected in Depdc7$^{-/-}$ oocytes.

We have added in the Result of the revised manuscript (line 158-162):

"To examine whether cytoplasmic actin was affected in *Depdc7*$^{-/-}$ oocytes, we assessed F-actin using phalloidin staining. Cytoplasmic F-actin intensity was not reduced; rather, it showed a slight but statistically significant increase (Appendix Fig. S2), indicating that DEPDC7 deficiency specifically disrupts cortical actin polarization without affecting overall cytoplasmic F-actin dynamics."

And we have also added corresponding discussion in the Discussion section of the revised manuscript. (line 301-310):

"It should be noted that our results support an essential role of *Depdc7*$^{-/-}$ in recruiting actin cap–associated proteins to the polarized cortical region, whereas the expression levels of these proteins appear unaffected (Fig. EV3C).

Furthermore, the non-polar cortical F-actin was largely intact, as stable subcortical F-actin could still be observed in *Depdc7*$^{-/-}$ oocytes. To assess cytoplasmic F-actin dynamics, we quantified GV-stage cytoplasmic F-actin intensity and found that it was largely unchanged in *Depdc7*$^{-/-}$ oocytes (Appendix Fig. S2). Consistent with this, the spindle was still able to migrate toward the cortex during metaphase (movie EV1-4). Collectively, these observations indicate that DEPDC7 plays a unique role in actin cap assembly, specifically affecting F-actin organization at the polarized cortex while largely sparing global F-actin dynamics."

4. How Ran-GTP determined DEPDC7 localization, and how DEPDC7 regulated CDC42 to the actin cap are now explained. The potential mechanism should be explored to fill the gap.

Response: We thank the referee for this important suggestion. We agree that further elucidation of how Ran-GTP determines DEPDC7 localization and how DEPDC7 regulates CDC42 to the actin cap would strengthen the mechanistic depth of the study. To address this question, we performed a series of exploratory molecular experiments in both cultured cells and oocytes, including co-immunoprecipitation (IP) assays and live-cell imaging analyses. However, we did not obtain direct evidence supporting a physical interaction or a definitive molecular linkage between DEPDC7 and Ran or CDC42. These results suggest that the regulation may be indirect or mediated by additional intermediates that remain to be identified. We therefore acknowledge that the precise molecular mechanism underlying this pathway remains to be fully elucidated. We have now included this point as a limitation in the Discussion and highlighted it as an important direction for future investigation (line 353-360). Importantly, this does not alter our central conclusion that DEPDC7 is required for proper actin cap assembly during oocyte meiosis.

"Third, the precise molecular mechanism linking Ran-GTP signaling to DEPDC7 cortical localization and CDC42 recruitment remains unclear. We performed exploratory experiments, but did not obtain direct evidence of a physical interaction between DEPDC7 and Ran or CDC42. DEPDC7 may act downstream of Ran-GTP through indirect regulatory pathways or additional intermediates that remain to be identified. Further biochemical and mechanistic studies will be required to elucidate the molecular network connecting Ran-GTP signaling, DEPDC7, and CDC42-mediated actin remodeling during oocyte maturation."

5. The errors should be provided for the number of chromosomes Fig 6F.

Response: We thank the referee for the helpful suggestion. The original Figure 6F showed chromosome numbers as bars, but because all WT embryos (n = 19) were diploid and all Depdc7$^{-/-}$ embryos (n = 24) were triploid, the fixed bar heights could be misleading. To more clearly and accurately represent the data, we have replaced Figure 6F with a percentage-based bar graph, which directly shows that 100% of WT embryos are diploid and 100% of Depdc7$^{-/-}$ embryos are triploid. As all embryos within each group are identical, no error bars are included.

6. The spindle positioning for the actin cap formation in the diagram of Fig 8 is not correct. The spindle migrates to the cortex, and the pole is faced to the actin cap, but there is no rotation of spindle in late MI stage, since it only occurred after the polar body extrusion.

Response: Thanks for your comments. We have revised Figure 8 as suggested.

Dear Prof. Sang

Thank you for the submission of your revised manuscript to EMBO reports. We have now received the full set of referee reports that is copied below.

As you can see, the referees find that the study is significantly improved during revision and recommend publication. Before I can accept the manuscript, I need you to address some editorial points below:

- Please rename the "Data and materials availability" section to "Data Availability" and place it before the Acknowledgements.

- Please rename the "Competing interest" section to "Disclosure and Competing Interests Statement" and remove the "Competing interests" statement from the title page.

- Please rename the "Material and Methods" section to "Methods".

- Please include an approval number for the entitiy granting ethics approval and a description of housing conditions in the Methods section.

- Please include statements on blinding and randomization in the Methods section even if no blinding or randomization were done.

- Please remove the instructions from the Reagents and Tools Table.

- Please note that omics data needs to be depositied in a publicly available repository. The specific URL for the scRNA-seq dataset should be provided in the data availability statement, i.e., we need a link that resolves directly to the dataset. Please ensure that data listed in the Data Availability section becomes publicly accessible at this point, latest upon formal acceptance.

- Please fill out the Data Availability Section of the Author Checklist to reflect the scRNA-seq dataset.

- CRediT has replaced the traditional author contributions section because it offers a systematic machine readable author contributions format that allows for more effective research assessment. Please remove the Author Contributions section from the manuscript and use the free text boxes beneath each contributing author's name in our online systems to add specific details on the author's contribution. More information is available in our guide to authors.

- Please adjust the format of the reference list and of the in-text citations according to EMBO Journal format (alphabetical order rather than numerical and up to 10 author names in the reference list before et al). Please refer to our Guide to Authors for additional information on EMBO J reference format.

- Please make sure to all relevant funding information in the manuscript is also entered into our submission system. The funding information in the manuscript needs to be congruent with the info entered into our online system. The New Cornerstone Science Foundation (and the XPLORER PRIZE) and SANS Exploration should be entered in the system via More Funders option. The comments box should not be used, please remove the funders from there.

- Please adjust the in-text callouts for individual figures and figure panels: "Supplementary information" should not be used.

- Please upload Dataset EV1 as an excel file without zipping and provide a legend as an additional tab/sheet in the excel file.

- Please remove the author information from the title page of the Appendix file (we just need the title and table of contents). Please update the nomenclature in the file to Appendix Figure S1, etc.

- Please move the primer information from the Appendix file to the Reagents and Tools Table.

- Please please provide a legend for each movie. The legend should provided in a readme.txt files and zipped together with its corresponding movie such that there is a zip folder per movie uploaded.

- Please note that EMBO press papers are accompanied online by:
A) a short (2 sentences) summary of the findings and their significance,
B) 2-5 short bullet points highlighting the key results, and
C) a synopsis image in .jpg or .png format that is exactly 550 pixels wide and 300 pixels high. Please note that the text needs to be legible at the final size. Please upload this information along with your revised manuscript (the text for A and B should be provided in one separate Word file uploaded as Cover Art/Synopsis Image).

- Our production/data editors have asked you to clarify several points in the figure legends (see below). Please incorporate these changes in the manuscript and return the revised file with tracked changes with your final manuscript submission.
- Please note that the exact p values are not provided in the legends of figures 2D, G; 3D, 7B, EV2 E, EV4 C
- Please note that information related to n is missing in the legends of figures 1E, EV4 C, D, and Fig S2B
- Please note that the error bars are not defined in the legend of figure EV2 C
- Please note that the dashed borders is not defined in the legend of figures 7C, EV2 F.This needs to be rectified.

We look forward to seeing a final version of your manuscript as soon as possible.

Sincerely,

Kurt Weir
Editor
EMBO Reports

Referee #1:

The revised manuscript addresses my concerns, and the additional experiments and revisions strengthen the manuscript.

Referee #2:

The authors have provided a number of additional experiments and analyses which fully answer my concerns. I have no further request and I recommend publication of this revised manuscript.

Referee #3:

The authors basically addressed my concerns.

As you can see, the referees find that the study is significantly improved during revision and recommend publication. Before I can accept the manuscript, I need you to address some editorial points below:

Thank you very much for your positive evaluation of our revised manuscript and for the helpful editorial comments. We have carefully addressed all of the points raised and revised the manuscript accordingly. In addition, we have corrected several minor textual and formatting issues throughout the manuscript to improve clarity and consistency. A revised version with tracked changes has been included with the submission.

Please find our point-by-point responses below.

- Please rename the "Data and materials availability" section to "Data Availability" and place it before the Acknowledgements.

Response: The section title has been changed to “Data Availability” and moved before the Acknowledgements section as requested (line 524).

- Please rename the "Competing interest" section to "Disclosure and Competing Interests Statement" and remove the "Competing interests" statement from the title page.

Response: The section title has been changed to “Disclosure and Competing Interests Statement”, and we have removed "Competing interests" statement from the title page (line 531).

- Please rename the "Material and Methods" section to "Methods".

Response: The section title has been changed to “Methods” (line 389).

- Please include an approval number for the entitiy granting ethics approval and a description of housing conditions in the Methods section.

Response: We have added the description of housing conditions and approval number in the Methods section (line 396-401).

- Please include statements on blinding and randomization in the Methods section even if no blinding or randomization were done.

Response: As requested, we have added explicit statements regarding blinding and randomization to the Methods section (under the "Statistical analysis" sub-section). We have clarified that no blinding or randomization was performed for

the experiments in this study. (line 520)

- Please remove the instructions from the Reagents and Tools Table.

Response: We have removed the instructions from the Reagents and Tools Table.

- Please note that omics data needs to be depositied in a publicly available repository. The specific URL for the scRNA-seq dataset should be provided in the data availability statement, i.e., we need a link that resolves directly to the dataset. Please ensure that data listed in the Data Availability section becomes publicly accessible at this point, latest upon formal acceptance.

Response: The scRNA-seq dataset has been deposited in the Genome Sequence Archive (GSA) repository with the accession number CRA042768 (https://ngdc.cncb.ac.cn/gsa/search?searchTerm=CRA042768). We also have updated the Data Availability section. (line 524)

- Please fill out the Data Availability Section of the Author Checklist to reflect the scRNA-seq dataset.

Response: The Data Availability section of the Author Checklist has been updated to include the scRNA-seq dataset information.

- CRediT has replaced the traditional author contributions section because it offers a systematic machine readable author contributions format that allows for more effective research assessment. Please remove the Author Contributions section from the manuscript and use the free text boxes beneath each contributing author's name in our online systems to add specific details on the author's contribution. More information is available in our guide to authors.

Response: Author Contributions section has been removed, and the contribution details for each author have been provided in the online submission system as requested.

- Please adjust the format of the reference list and of the in-text citations according to EMBO Journal format (alphabetical order rather than numerical and up to 10 author names in the reference list before et al). Please refer to our Guide to Authors for additional information on EMBO J reference format.

Response: We have thoroughly adjusted the format of both the in-text citations and the reference list to comply with the EMBO Journal style.

- Please make sure to all relevant funding information in the manuscript is also entered into our

submission system. The funding information in the manuscript needs to be congruent with the info entered into our online system. The New Cornerstone Science Foundation (and the XPLORER PRIZE) and SANS Exploration should be entered in the system via More Funders option. The comments box should not be used, please remove the funders from there.

Response: The funding information in the manuscript and the online submission system has been updated for consistency. The New Cornerstone Science Foundation (including the XPLORER PRIZE) and SANS Exploration have been entered via the "More Funders" option and removed from the comments box as requested.

- Please adjust the in-text callouts for individual figures and figure panels: "Supplementary information" should not be used.

Response: The wording referring to "Supplementary information" has been revised for consistency with the journal format requirements.

- Please upload Dataset EV1 as an excel file without zipping and provide a legend as an additional tab/sheet in the excel file.

Response: The format of Dataset EV1 has been updated as an Excel file without zipping, and the legend has been added as an additional worksheet in the file as requested.

- Please remove the author information from the title page of the Appendix file (we just need the title and table of contents). Please update the nomenclature in the file to Appendix Figure S1, etc.

Response: Author information has been removed from the Appendix title page, and all figure labels have been updated to "Appendix Figure S1, S2, etc." throughout the file.

- Please move the primer information from the Appendix file to the Reagents and Tools Table.

Response: The primer information has been moved from the Appendix (formerly Table S1) to the Reagents and Tools Table as requested.

- Please please provide a legend for each movie. The legend should provided in a readme.txt files and zipped together with its corresponding movie such that there is a zip folder per movie uploaded.

Response: A separate legend has been prepared for each movie in a readme.txt

file. Each movie file and its corresponding readme.txt have been zipped together into individual folders

- Please note that EMBO press papers are accompanied online by:

A) a short (2 sentences) summary of the findings and their significance,

B) 2-5 short bullet points highlighting the key results, and

C) a synopsis image in .jpg or .png format that is exactly 550 pixels wide and 300 pixels high. Please note that the text needs to be legible at the final size. Please upload this information along with your revised manuscript (the text for A and B should be provided in one separate Word file uploaded as Cover Art/Synopsis Image).

Response: The requested summary, highlights, and synopsis image have been prepared and uploaded with the revised submission. The text for items A and B has been provided in a separate Word file uploaded as "Cover Art/Synopsis Image" as requested.

- Our production/data editors have asked you to clarify several points in the figure legends (see below). Please incorporate these changes in the manuscript and return the revised file with tracked changes with your final manuscript submission.

- Please note that the exact p values are not provided in the legends of figures 2D, G; 3D, 7B, EV2 E, EV4 C

Response: The exact p values have now been added to the legends of Figures 2D, G; 3D; 7B; EV2E; and EV4C as requested.

- Please note that information related to n is missing in the legends of figures 1E, EV4 C, D, and Fig S2B

Response: Information related to n has now been added to the legends of Figures 1E, EV4C, EV4D, and Fig S2B as requested.

- Please note that the error bars are not defined in the legend of figure EV2 C

Response: The error bars have been defined in the legend of figure EV2 C.

- Please note that the dashed borders is not defined in the legend of figures 7C, EV2 F.This needs to be rectified.

Response: The dashed borders have been defined in the legend of figures 7C, EV2 F.

Prof. Qing Sang
Fudan University
Institutes of Biomedical Sciences
Dongan Road 131
Shanghai, Shanghai 200032
China

Dear Prof. Sang,

I am pleased to inform you that your manuscript has been accepted for publication in EMBO reports. Your manuscript will be processed for publication by EMBO Press. It will be copy edited and you will receive page proofs prior to publication. Please note that you will be contacted by Springer Nature Author Services to complete licensing and payment information.

You may qualify for financial assistance for your publication charges - either via a Springer Nature fully open access agreement or an EMBO initiative. Check your eligibility: https://link.springer.com/journal/44319/how-to-publish-with-us

Should you be planning a Press Release on your article, please notify the production team via this form in order to coordinate publication and release dates: https://forms.cloud.microsoft/e/eH4A8JVP6g

If you have any questions, please do not hesitate to contact the Editorial Office. Thank you for your contribution to EMBO Reports.

Yours sincerely,

Kurt Weir
Editor
EMBO Reports

>>> Please note that it is EMBO Reports policy for the transcript of the editorial process (containing referee reports and your response letter) to be published as an online supplement to each paper. If you do NOT want this, you will need to inform the Editorial Office via email immediately. More information is available here: https://link.springer.com/partners/embo-press/editorial-policies#Peer%20review

EMBO Press Author Checklist

Corresponding Author Name: Qing Sang
Journal Submitted to: EMBO reports
Manuscript Number: EMBOR-2025-63003

USEFUL LINKS FOR COMPLETING THIS FORM
The EMBO Journal - Author Guidelines
EMBO Reports - Author Guidelines
Molecular Systems Biology - Author Guidelines
EMBO Molecular Medicine - Author Guidelines

Reporting Checklist for Life Science Articles (updated January
This checklist is adapted from Materials Design Analysis Reporting (MDAR) Checklist for Authors. MDAR establishes a minimum set of requirements in transparent reporting in the life sciences (see Statement of Task: 10.31222/osf.io/9sm4x). Please follow the journal's guidelines in preparing your
Please note that a copy of this checklist will be published alongside your article.

Abridged guidelines for figures
1. Data
The data shown in figures should satisfy the following conditions:
- the data were obtained and processed according to the field's best practice and are presented to reflect the results of the experiments in an accurate and unbiased manner.
- ideally, figure panels should include only measurements that are directly comparable to each other and obtained with the same assay.
- plots include clearly labeled error bars for independent experiments and sample sizes. Unless justified, error bars should not be shown for technical
- if n<5, the individual data points from each experiment should be plotted. Any statistical test employed should be justified.
- Source Data should be included to report the data underlying figures according to the guidelines set out in the authorship guidelines on Data

2. Captions
Each figure caption should contain the following information, for each panel where they are relevant:
- a specification of the experimental system investigated (eg cell line, species name).
- the assay(s) and method(s) used to carry out the reported observations and measurements.
- an explicit mention of the biological and chemical entity(ies) that are being measured.
- an explicit mention of the biological and chemical entity(ies) that are altered/varied/perturbed in a controlled manner.
- the exact sample size (n) for each experimental group/condition, given as a number, not a range;
- a description of the sample collection allowing the reader to understand whether the samples represent technical or biological replicates (including how many animals, litters, cultures, etc.).
- a statement of how many times the experiment shown was independently replicated in the laboratory.
- definitions of statistical methods and measures:
  - common tests, such as t-test (please specify whether paired vs. unpaired), simple χ2 tests, Wilcoxon and Mann-Whitney tests, can be unambiguously identified by name only, but more complex techniques should be described in the methods section;
  - are tests one-sided or two-sided?
  - are there adjustments for multiple comparisons?
  - exact statistical test results, e.g., P values = x but not P values < x;
  - definition of 'center values' as median or average;
  - definition of error bars as s.d. or s.e.m.

Please complete ALL of the questions below.
Select "Not Applicable" only when the requested information is not relevant for your study.

Materials

Newly Created Materials	Information included in the manuscript?	In which section is the information available? (Reagents and Tools Table, Materials and Methods, Figures, Data Availability Section)
New materials and reagents need to be available; do any restrictions apply?	Not Applicable	

Antibodies	Information included in the manuscript?	In which section is the information available? (Reagents and Tools Table, Materials and Methods, Figures, Data Availability Section)
For **antibodies** provide the following information:<br>- Commercial antibodies: RRID (if possible) or supplier name, catalogue number and or/clone number<br>- Non-commercial: RRID or citation	Yes	Reagents and Tools Table, Materials and Methods

DNA and RNA sequences	Information included in the manuscript?	In which section is the information available? (Reagents and Tools Table, Materials and Methods, Figures, Data Availability Section)
**Short novel DNA or RNA including primers, probes**: provide the sequences.	Yes	Reagents and Tools Table

Cell materials	Information included in the manuscript?	In which section is the information available? (Reagents and Tools Table, Materials and Methods, Figures, Data Availability Section)
**Cell lines:** Provide species information, strain. Provide accession number in repository **OR** supplier name, catalog number, clone number, and/**OR** RRID.	Not Applicable	
**Primary cultures:** Provide species, strain, sex of origin, genetic modification status.	Not Applicable	
Report if the cell lines were recently **authenticated** (e.g., by STR profiling) and tested for mycoplasma contamination.	Not Applicable	

Experimental animals	Information included in the manuscript?	In which section is the information available? (Reagents and Tools Table, Materials and Methods, Figures, Data Availability Section)
**Laboratory animals or Model organisms:** Provide species, strain, sex, age, genetic modification status. Provide accession number in repository **OR** supplier name, catalog number, clone number, **OR** RRID.	Yes	Reagents and Tools Table, Materials and Methods
**Animal observed in or captured from the field:** Provide species, sex, and age where possible.	Not Applicable	
Please detail **housing and husbandry conditions**.	Yes	Materials and Methods

Plants and microbes	Information included in the manuscript?	In which section is the information available? (Reagents and Tools Table, Materials and Methods, Figures, Data Availability Section)
**Plants:** provide species and strain, ecotype and cultivar where relevant, unique accession number if available, and source (including location for collected wild specimens).	Not Applicable	
**Microbes:** provide species and strain, unique accession number if available, and source.	Not Applicable	

Human research participants	Information included in the manuscript?	In which section is the information available? (Reagents and Tools Table, Materials and Methods, Figures, Data Availability Section)
If collected and within the bounds of privacy constraints report on age, sex and gender or ethnicity for all study participants.	Not Applicable	

Core facilities	Information included in the manuscript?	In which section is the information available? (Reagents and Tools Table, Materials and Methods, Figures, Data Availability Section)
If your work benefited from core facilities, was their service mentioned in the acknowledgments section?	Yes	Acknowledgments

Design

Study protocol	Information included in the manuscript?	In which section is the information available? (Reagents and Tools Table, Materials and Methods, Figures, Data Availability Section)
If study protocol has been **pre-registered, provide DOI in the manuscript**. For clinical trials, provide the trial registration number **OR** cite DOI.	Not Applicable	
Report the **clinical trial registration number** (at ClinicalTrials.gov or equivalent), where applicable.	Not Applicable	

Laboratory protocol	Information included in the manuscript?	In which section is the information available? (Reagents and Tools Table, Materials and Methods, Figures, Data Availability Section)
Provide DOI OR other citation details if **external detailed step-by-step protocols** are available.	Not Applicable	

Experimental study design and statistics	Information included in the manuscript?	In which section is the information available? (Reagents and Tools Table, Materials and Methods, Figures, Data Availability Section)
Include a statement about **sample size** estimate even if no statistical methods were used.	Yes	Materials and Methods, Figure Legends
Were any steps taken to minimize the effects of subjective bias when allocating animals/samples to treatment (e.g. **randomization procedure**)? If yes, have they been described?	Yes	Materials and Methods
Include a statement about **blinding** even if no blinding was done.	Yes	Materials and Methods
Describe **inclusion/exclusion criteria** if samples or animals were excluded from the analysis. Were the criteria pre-established?<br><br>If sample or data points were omitted from analysis, report if this was due to attrition or intentional exclusion and provide justification.	Yes	Materials and Methods
For every figure, are **statistical tests** justified as appropriate? Do the data meet the assumptions of the tests (e.g., normal distribution)? Describe any methods used to assess it. Is there an estimate of variation within each group of data? Is the variance similar between the groups that are being statistically compared?	Yes	Materials and Methods, Figure Legends

Sample definition and in-laboratory replication	Information included in the manuscript?	In which section is the information available? (Reagents and Tools Table, Materials and Methods, Figures, Data Availability Section)
In the figure legends: state number of times the experiment was **replicated** in laboratory.	Yes	Figure Legends
In the figure legends: define whether data describe **technical or biological replicates**.	Yes	Figure Legends

Ethics

Ethics	Information included in the manuscript?	In which section is the information available? (Reagents and Tools Table, Materials and Methods, Figures, Data Availability Section)
Studies involving **human participants**: State details of **authority granting ethics approval** (IRB or equivalent committee(s), provide reference number for approval.	Not Applicable	
Studies involving **human participants**: Include a statement confirming that **informed consent** was obtained from all subjects and that the experiments conformed to the principles set out in the WMA Declaration of Helsinki and the Department of Health and Human Services Belmont Report.	Not Applicable	
Studies involving **human participants:** For publication of **patient photos**, include a statement confirming that consent to publish was obtained.	Not Applicable	
Studies involving experimental **animals**: State details of **authority granting ethics approval** (IRB or equivalent committee(s), provide reference number for approval. Include a statement of compliance with ethical regulations.	Yes	Materials and Methods
Studies involving **specimen and field samples:** State if relevant **permits** obtained, provide details of authority approving study; if none were required, explain why.	Not Applicable	

Dual Use Research of Concern (DURC)	Information included in the manuscript?	In which section is the information available? (Reagents and Tools Table, Materials and Methods, Figures, Data Availability Section)
Could your study fall under dual use research restrictions? Please check biosecurity documents and list of **select agents and toxins** (CDC): https://www.selectagents.gov/sat/list.htm	Not Applicable	
If you used a select agent, is the security level of the lab appropriate and reported in the manuscript?	Not Applicable	
If a study is subject to dual use research of concern regulations, is the name of the **authority granting approval and reference number** for the regulatory approval provided in the manuscript?	Not Applicable	

Reporting

The MDAR framework recommends adoption of discipline-specific guidelines, established and endorsed through community initiatives. Journals have their own policy about requiring specific guidelines and recommendations to complement MDAR.

Adherence to community standards	Information included in the manuscript?	In which section is the information available? (Reagents and Tools Table, Materials and Methods, Figures, Data Availability Section)
State if relevant guidelines or checklists (e.g., **ICMJE, MIBBI, ARRIVE, PRISMA**) have been followed or provided.	Not Applicable	
For **tumor marker prognostic studies,** we recommend that you follow the **REMARK** reporting guidelines (see link list at top right). See author guidelines, under 'Reporting Guidelines'. Please confirm you have followed these guidelines.	Not Applicable	
For **phase II and III randomized controlled trials**, please refer to the **CONSORT** flow diagram (see link list at top right) and submit the CONSORT checklist (see link list at top right) with your submission. See author guidelines, under 'Reporting Guidelines'. Please confirm you have submitted this list.	Not Applicable	

Data Availability

Data availability	Information included in the manuscript?	In which section is the information available? (Reagents and Tools Table, Materials and Methods, Figures, Data Availability Section)
Have **primary datasets** been deposited according to the journal's guidelines (see 'Data Deposition' section) and the respective accession numbers provided in the Data Availability Section?	Yes	Data availability Section
Were **human clinical and genomic datasets** deposited in a public access-controlled repository in accordance to ethical obligations to the patients and to the applicable consent agreement?	Not Applicable	
Are **computational models** that are central and integral to a study available without restrictions in a machine-readable form? Were the relevant accession numbers or links provided?	Not Applicable	
If publicly available data were reused, provide the respective **data citations in the reference list**.	Not Applicable	
